# Exosome-sheathed ROS-responsive nanogel to improve targeted therapy in perimenopausal depression

**DOI:** 10.1186/s12951-023-02005-y

**Published:** 2023-08-08

**Authors:** Yue Hu, Min Zhao, Hui Wang, Yang Guo, Xiaolan Cheng, Tong Zhao, Hanqing Wang, Yafeng Zhang, Yong Ma, Weiwei Tao

**Affiliations:** 1 Jiangsu CM Clinical Innovation Center of Degenerative Bone & Joint Disease, Wuxi TCM Hospital Affiliated to Nanjing University of Chinese Medicine, 8 Zhongnan West Road, Wuxi, 214071 China; 2 School of Chinese Medicine, School of Integrated Chinese and Western Medicine, Nanjing University of Chinese Medicine, 138 Xianlin Road, Nanjing, 210023 China; 3 School of pharmacology, Nanjing University of Chinese Medicine, 138 Xianlin Road, Nanjing, 210023 China; 4 School of pharmacology, Ningxia Medical University, 1160 Shengli Street, Xingqing District, Yinchuan, China; 5 Jiangsu Collaborative Innovation Center of Chinese Medicinal Resources Industrialization, Nanjing University of Chinese Medicine, 138 Xianlin Road, Nanjing, 210023 China

**Keywords:** Exosome, Nanogel, Perimenopausal Depression, PACAP, Estrogen

## Abstract

The development of natural membranes as coatings for nanoparticles to traverse the blood-brain barrier (BBB) presents an effective approach for treating central nervous system (CNS) disorders. In this study, we have designed a nanogel loaded with PACAP and estrogen (E2), sheathed with exosomes and responsive to reactive oxygen species (ROS), denoted as HA NGs@exosomes. The objective of this novel design is to serve as a potent drug carrier for the targeted treatment of perimenopausal depression. The efficient cellular uptake and BBB penetration of HA NGs@exosomes has been demonstrated in vitro and in vivo. Following intranasal intervention with HA NGs@exosomes, ovariectomized mice under chronic unpredictable mild stress (CUMS) have shown improved behavioral performance, indicating that HA NGs@exosomes produced a rapid-onset antidepressant effect. Moreover, HA NGs@exosomes exhibit notable antioxidant and anti-inflammatory properties and may regulate the expression of pivotal proteins in the PACAP/PAC1 pathway to promote synaptic plasticity. Our results serve as a proof-of-concept for the utility of exosome-sheathed ROS-responsive nanogel as a promising drug carrier for the treatment of perimenopausal depression.

**Graphical Abstract:**

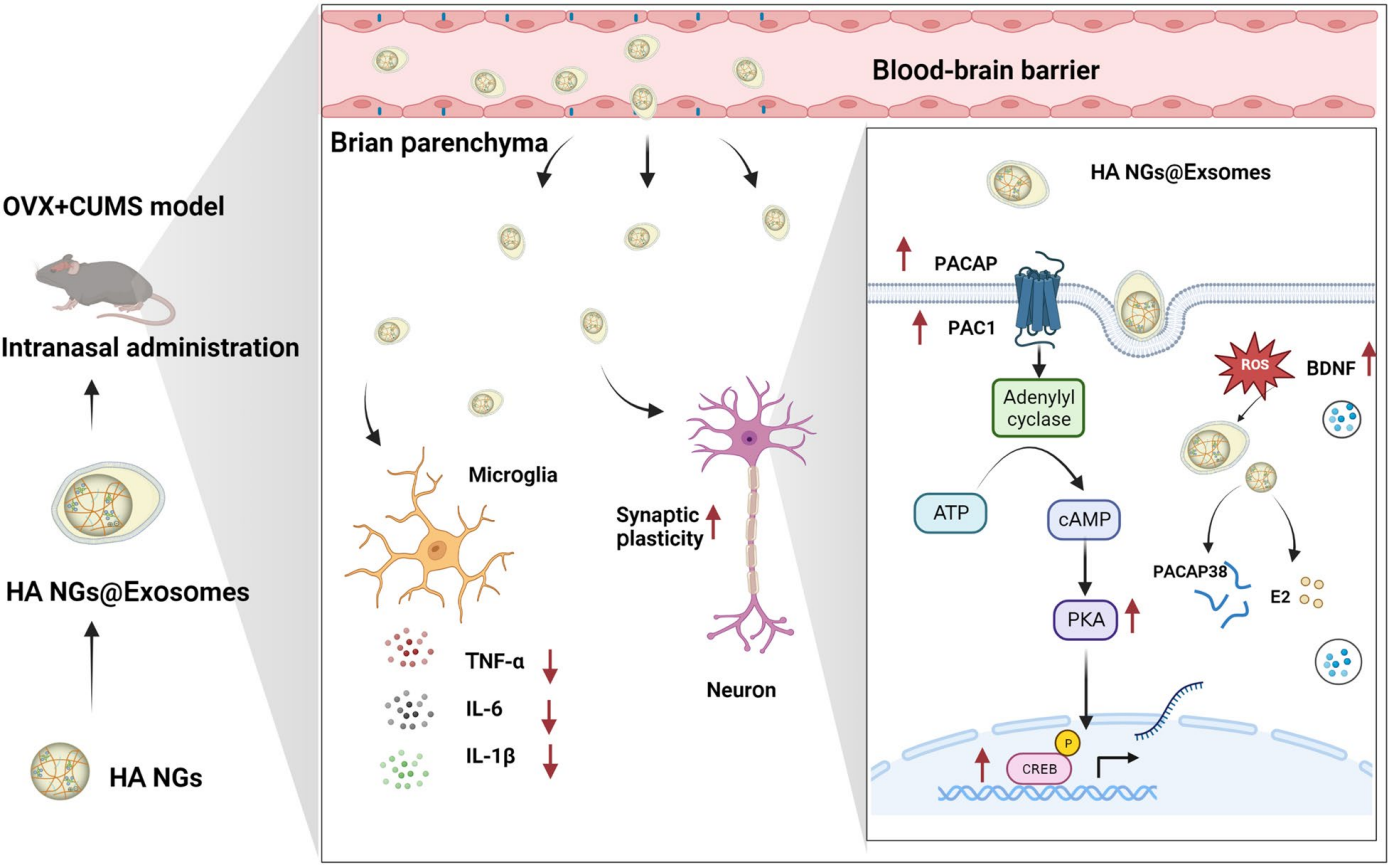

**Supplementary Information:**

The online version contains supplementary material available at 10.1186/s12951-023-02005-y.

## Introduction

The incidence of depressive disorder in perimenopausal women is reportedly higher than that of pre- or postmenopausal women [1, 2]. Perimenopausal depression is characterized by sleep and mood disturbances, such as melancholia and agitation, and the risk of suicide appears to be increasing annually [3]. The conventional approach to treating perimenopausal depression is a combination of antidepressants and estrogen (E2) replacement therapy [4, 5]. Studies have indicated that E2 therapy has favorable effects on mood in perimenopausal women [6]. Furthermore, E2 might enhance the efficacy of antidepressants and decrease the response time [7]. Nevertheless, the use of estradiol treatment has been associated with side effects, including bleeding [8], coronary heart disease [9], and an elevated risk of breast cancer [10]. These side effects may be attributable to hormonal level fluctuations in the circulatory system [11]. In addition, the pathological process of depression is closely associated with the elevated level of reactive oxygen species (ROS) [12, 13]. The increased level of ROS and exhaustion of antioxidative defenses are responsible for the altered brain microenvironment and structure [14]. Therefore, there is an urgent need to develop ROS-responsive, efficacious, and more stable E2 treatment systems with improved safety profiles.

Pituitary adenylate cyclase-activating polypeptide (PACAP), an endogenous neuropeptide with 38-amino acid, was originally isolated from ovine hypothalamic extracts in 1989 [15]. It is a member of the highly conserved vasoactive intestinal polypeptide-secretin-glucagon superfamily and is widely distributed in the brain and peripheral organs [16–18]. According to the latest updates to the Human Protein Atlas program database, the expression of PACAP (encoded by the *ADCYAP1* gene) predominantly occurs in granulocytes and excitatory neurons [19]. PACAP demonstrates a high affinity for its receptor, PAC1, which facilitates the execution of additional functions within the central nervous system (CNS). As a neuromodulator, neurotrophic factor, or neurotransmitter, PACAP is involved in pleiotropic functions in CNS, such as promoting neurogenesis [20, 21], enhancing the development of hypersensitivity responses [22], anti-infection [23, 24], inhibiting neuroinflammation [25], and regulating certain stress responses [26–28]. Despite this, the exact mechanisms underlying PACAP’s influence on depression and its potential therapeutic effects remain largely undiscovered.

Nanoparticles-based drug delivery systems have shown promising potential in enhancing drug delivery across the blood-brain barrier (BBB) [29, 30]. Nanoparticles have advantages in controlling of drug release and modifying drug pharmacokinetics. Moreover, nanoparticles can be multifunctional and possess different targets, such as antibodies, peptides, or other biomolecules [31, 32]. Additionally, nanoparticles can be encapsulated by natural biomaterials, such as cell membranes [33], to display better biocompatibility, targeting capacity, and prolonged circulation [34–36]. Among the vinous biomaterials, exosomes, small extracellular vesicles secreted by different types of single cells or integrating cells [37, 38], exhibit excellent biocompatibility, circulating stability, lower toxicity, and non-immunogenicity [39–41]. Additionally, the use of exosome-sheathed nanoparticles can enhance pharmacokinetic behavior, including blood circulation, biodistribution, and tissue targeting [42]. Furthermore, these nanoparticles facilitate nano-bio interactions and offer desirable functionality within the tissue [43]. Exosomes have been widely utilized to deliver small molecules, proteins, and nucleic acids across the BBB to treat inflammatory and neurodegenerative disorders [44–47]. However, the effectiveness of exosome-biomimetic nanoparticles in treating depressive disorders lacks sufficient evidence. Nanogels are three-dimensional hydrogels crosslinked hydrophilic polymeric networks [48], which exhibit the properties of both hydrogels and nanoparticles. In the biomedical field, nanogels are considered innovative materials and promising candidates for nanocarriers in the delivery of active substances to the CNS [49, 50].

In this study, we synthesized an exosome-shielded degradable nanogel (NG), known as hyaluronic acid (HA) NGs@exosomes loaded with PACAP and E2 (HA NGs@exosomes), for targeted delivery of peptides/drugs and improved treatment of perimenopausal depression. The NGs are reactive oxygen species (ROS)-responsive, meaning that they can rapidly respond to oxidative stimuli (e.g., H_2_O_2_), and subsequently degrade for drug release. These features of HA NGs@exosomes can result in a higher degree of enrichment in vivo and more precise drug release in the brain. Herein, our study provides a novel approach for perimenopausal depression therapy using exosome-sheathed nanogel as drug carriers for the efficient delivery of anti-perimenopausal and anti-depressive drugs.

## Materials and methods

### Regents and materials

All reagents were of analytical quality. AG 50 W-X8 resin was purchased from Bio-Rad (USA). PACAP was purchased from Med Chem Express (NewJersey, USA). Sodium hyaluronate (MW 7.46 kDa), Ethanol, Dimethyl sulfoxide (DMSO), tetrabutylammonium fluoride hydrate (TBA-F), 11-amino-1-undecanethiol hydrochloride (AT), N-hydroxysulfosuccinimide (NHS), 1-ethyl-3-[3-dimethylaminopropyl] carbodiimide hydrochloride (EDC), Estrogen was purchased from Aldrich Chemical Co. All solvents were of analytical quality. LPS (L6529) was purchased from Sigma-Aldrich, St. Louis, MO (USA), dimethyl amiloride (DMA) was purchased from Sigma-Aldrich (St Louis, MO, USA), DIO, ionomycin, were purchased from Beyotime Biotechnology (Shanghai, China).

### Preparation of HA-AT

First, commercially obtained sodium hyaluronate was modified by a long thiolated alkyl chain to produce an amphiphilic conjugate. To solubilize sodium hyaluronate in DMSO, the sodium ion of HA was exchanged with TBA-F using an AG 50 W-X8 cation exchange resin according to the reported method [51]. The resulting HA-TBA solution was lyophilized and the obtained fluffy white substance was stored at room temperature. In the presence of EDC and NHS, Hydrophobic 11-amino-1-undecanethiol hydrochloride (AT) was attached to HA-TBA. Specifically, HA-TBA was dissolved in DMSO containing 1% (w/v) EDC and NHS. Then, AT dissolved in anhydrous DMSO was added to the reaction mixture, and stirred for 24 h at room temperature. Finally, the solution was dialyzed (MWCO 1000 Da) with a proper concentration of NaCl (the weight of NaCl has been calculated according to the volume of liquid samples). The total weight obtained after freeze-drying is subtracted from the calculated NaCl content to get actual sample content as 1 mg/mL. After 3 days, a white cotton-like material was obtained, corresponding to the HA-AT conjugate.

### Preparation of HA nanogels (HA NGs)

First, CY5.5-labeled PACAP38 was prepared by coupling N-hydroxysuccinimide (NHS) and 1-ethyl-3-(3-dimethyl aminopropyl)-carbodiimide (EDC). Briefly, PACAP38 was mixed with NHS and EDC in 4-(2-hydroxyethyl)-1-piperazine ethane sulfonic acid (HEPES, pH 5.5) buffer for 30 min. Afterward, CY5.5 was added to the reaction mixture and stirred for 3 h. The CY5.5-labeled PACAP38 was harvested by dialysis. For the preparation of nanogels, HA-AT has dispersed in PBS (7.4) solution at 1.0 mg/mL, to which E2 (0.5 mg/mL in ethanol) and PACAP38 (200 µg/mL in PBS) was added and stirred overnight. The resulting solution was filtered through a membrane of 0.22 mm pore size and HA NGs were isolated by centrifugation at 12,000 g. Finally, HA NGs was dispersed in deionized water at -4℃ for further experiment.

### Loading capacity (LC) and encapsulation efficiency (EE) of PACAP and E2

At the given concentrations (HA-AT: 1.0 mg/mL; E2: 0.5 mg/mL; PACAP38: 200 µg/mL), the EE of PACAP and E2 were measured (Table 1). Briefly, the total and residual PACAP was stained with Coomassie Blue and analyzed by UV-vis at the absorbance of 610 nm. In terms of E2, the quantification was determined by HPLC before and after the loading. The proportion of HA-AT: E2: TACAP = 1:0.5:0.2, the LC of PACAP: (0.2*0.7018)/(1 + 0.2) = 0.117; LC of E2: (0.5*0.4812)/(1 + 0.5) = 0.16.

### Preparation and identification of the exosomes

Approximately 6 × 10^5^ Raw264.7 cells were cultured in Dulbecco’s Modified Eagle Medium (DMEM) without fetal bovine serum (FBS) for three days. Exosome preparation was conducted as previously described [52]. Conditioned media (CM) was obtained from the supernatant of the culture medium after spinning at 400 × g for 10 min and 2,000 × g for 20 min. The CM was subjected to ultracentrifugation at 100,000 × g for 3 h. Subsequently, CM was concentrated to a volume of 1 mL using an Amicon Ultrafilter device following the manufacturer’s instructions. The collected pellets were finally resuspended in CellLyticM Cell Lysis Reagent (Sigma, St. Louis, Missouri, USA) and subjected to western blotting. The primary antibodies used included anti-Synt1 (ab133267, Abcam, Cambridge, UK), anti-CD81 (ab79559, Abcam, Cambridge, UK), anti-CD63 (ab216130, Abcam, Cambridge, UK) and anti-ALIX (ab275377, Abcam, Cambridge, UK). All primary antibodies were diluted to 1:2000.

### Preparation and confirmation of exosomes sheathed on HA NGs (HA NGs@exosomes)

#### Preparation

HA NGs mixed with exosomes were ultra-sounded for 20 min and then incubated at 37 °C for 30 min to allow exosomes to disassemble and reassemble on the NGs.

#### Yield

The fresh medium with exosomes was incubated with 200 µgmL^− 1^ HA NGs for 6 h and rinsed with PBS twice. Then, 15 nM DMA or 10 µM ionomycin was added. After 16 h incubation, the supernatants were collected, centrifuged at 5000 g for 15 min to remove debris, and then centrifuged at 20,000 g for 30 min. The content of HA NGs@exosomes was measured using ICP-OES system (Optima 4300 DV, PerkinElmer, USA).

#### Confirmation

Exosomes or HA NGs were stained with 20 µM DiO or CY5.5 for 45 min, respectively, centrifuged at 100,000 g for 60 min, and then washed three times with water. The labeled exosomes and HA NGs were used for the synthesis of HA NGs@exosomes and were observed by transmission electron microscopy (TEM) microscopy (Tecnai G2-20, FEI Corp., Netherlands). The DiO fluorescence was detected at the excitation wavelength of 488 nm and the emission range of 500–520 nm. The HA NGs was detected at the excitation wavelength of 675 nm and the emission range of 694–710 nm. The colocalization of DiO and HA NGs was observed by laser scanning confocal microscopy (Leica TCS-SP8 STED, Wetzlar, Germany). HA NGs@exosomes were lysed in RIPA lysis buffer and then subjected to western blot analysis. The primary antibodies were: anti-calnexin (ab133615, Abcam, Cambridge, UK), anti-CD9 (ab236630, Abcam, Cambridge, UK), anti-CD63 (ab216130, Abcam, Cambridge, UK), anti-TSG101 (ab125011, Abcam, Cambridge, UK). All primary antibodies were diluted to 1:2000.

### Characterization of HA NGs@exosomes

The hydrodynamic diameter and zeta potential of nanomaterials were determined by Dynamic light scattering (DLS) (ZetaSizer ZS90, Malvern Instruments Ltd., Worcestershire, UK). For DLS, NGs@exosomes incubating in PBS with or without 10% FBS for 6 days before measure. The size was measured in PBS and the zeta-potential was measured in 10 mM NaCl at 23 ℃ with a 173℃ scattering angle at least triplicates. To test the degradation behavior of HA NGs@exosomes, they were measured in PBS at 37 °C, 4 °C, and − 80 °C for 12 consecutive days. The morphology and size of HA NGs@exosomes were observed by TEM (Tecnai G2-20, FEI Corp., Netherlands). Images were obtained by laser scanning microscopy (Olympus IX 70 inverted microscope).

### In vitro release of E2 and PACAP from HA NGs

The release profiles of E2 and PACAP from HA NGs were evaluated by incubating with different concentrations of H_2_O_2_ in PBS buffer (pH 7.4) at 37 ℃ over prearranged time intervals. The released E2 in supernatant solutions was quantified by high-performance liquid chromatography (HPLC), and PACAP was stained with Coomassie Blue and quantified by UV-vis at 610 nm.

### Ethics statement

The protocols were carried out in accordance with the principles for laboratory animals (U.K. Animals Act and associated guidelines, 1986). All experiments involving animals were conducted according to the ethical policies and procedures approved by the Institutional Animal Care and Use Committee at Nanjing University of Chinese medicine (No. 202112A046), Nanjing University of Chinese medicine, China.

### Cell culture and treatment

Primary cortical neurons and microglia were cultured as previously described [53, 54]. Briefly, neurons were generated from the cortex of E18 C57BL/6 mouse embryos. The cortical tissues were harvested and digested enzymatically into cell suspensions. Then, the washed cells were resuspended with DMEM containing 10% FBS (Gibco, USA) and 2% B27 (Gibco, USA), and seeded onto poly-l-lysine-coated cell culture plates. To prepare primary microglia, cells were isolated from the brain of newborn 1 d C57BL/6 mouse pups. After removing the meninges, the tissue was separated into individual cell suspensions using mechanical disruption and trypsinization. Cells were then resuspended for 10 min and cultured in DMEM/F-12 (1:1) medium containing 10% FBS (Gibco, USA) and 1% penicillin/streptomycin. On the 8th day of culture, microglia were recovered by proteolysis using 0.05% trypsin. All cells were maintained in a humidified incubator with 95% air and 5% CO_2_ atmosphere at 37 °C. For the neuroinflammatory challenge model, the cells were treated with LPS (10 ng/ml) and incubated for 24 h, and then the subsequent experiments were performed as designed.

### In vitro cellular uptake assay

Primary neuronal cells received 10 ng/ml LPS stimulation for 12 h, then, HA NGs@exosomes with CY5.5-labeled PACAP and CY3-labeled HA NGs were added in the culture dish and incubated for 4 h. Afterward, the cells were washed with PBS 3 times, and the nuclear was stained with DAPI for 20 min. The images were captured under a confocal microscope (Leica TCS-SP8 STED, Wetzlar, Germany).

### ROS generation detection

To investigate the generation of ROS after different treatments, cells were seeded in 96-well plates. After stimulation with LPS (10 ng/ml), the cells were rinsed with PBS three times. 100 µL H2DCFDA was added into the cells with a concentration of 20 × 10^− 6^ m and incubated in the dark for 30 min at 37 °C. Then, the plates were measured by Multiskan MK3 microplate reader at the excitation wavelength of 488 nm, and the emission wavelength of 525 nm.

For Dihydroethidium (DHE) detection, brain tissues homogenized with PBS and centrifuged. Then, the supernatant was collected and incubated with 10 µM DHE at 37 °C for 30 min. The fluorescence was subsequently measured using a SpectraMax Microplate Reader (Molecular Devices, San Jose, CA) at 480 nm excitation and 576 nm emission wavelengths.

### Animal models and treatment

C57BL/6 6-week-old female mice (ChangZhou Cavens Laboratory Animal Center, ChangZhou, China) were housed five per cage under a temperature-controlled (22 ± 2 °C) environment and a 12-h light-dark cycle. Food and water were given ad libitum.

#### LPS-induced model

LPS-induced mice model was performed as previously described [55]. Mice were intraperitoneally injected with LPS (0.083 mg/kg) in saline at an interval of 1 h and then for another 24 h (or with saline as control) under anesthesia.

#### CUMS model

Specifically, the mice were subjected to discontinuously random mild stress including food deprivation for 24 h, water deprivation for 24 h, overnight illumination, moist bedding for 12 h, 45℃ cage tilting for 24 h, light/dark succession, and restraint for 2 h, etc. The overnight illumination was performed twice or three times per week. Sucrose preference test (SPT) was performed to evaluate the success of CUMS model.

#### OVX-CUMS model

OVX-CUMS model was conducted to refer to the reported method [56]. Specifically, for ovariectomy (OVX) surgery, mice were anesthetized with Pelltobarbitalum Natricum (50 mg/kg) via intraperitoneal injection (i.p.). Following small bilateral dorsal flank incisions, the ligation close to the uterus was performed and then the pink cauliflower-shaped ovary was removed immediately. Afterward, the surgical wounds of mice were sutured sequentially from muscle to skin. The ovary on the other side was also removed in the same manner. Another group of mice that underwent sham surgery with similar surgical and suture steps as described above but without ovary removal served as controls. All operated mice were given a two-week postoperative recovery period. Thereafter, the ovariectomized mice underwent CUMS for 14 days as described above. SPT was performed to evaluate the success of OVX-CUMS model.

#### Treatment

Following the successful completion of CUMS experiments, a total volume of 20 µl of HA NGs@exosomes (loaded with PACAP&E2) was administered intranasally. After 24 h, relevant experiments were conducted. In another series of studies, HA NGs@exosomes encapsulated with PACAP&Estrogen (E-HA PACAP&E2), HA NGs@exosomes encapsulated with PACAP only (E-HA PACAP), Free PACAP&E2, or their blank control vehicles (E-HA NC) were intranasally administrated to OVX-CUMS induced female mice at a dosage of 20 ul.

### In vivo biodistribution of HA NGs@exosomes

The biodistribution of HA NGs@exosomes was tested as previously described [57]. HA NGs@exosomes were labeled with a near-infrared lipophilic carbocyanine dye, DiR. Mice received DiR-HA NGs@exosomes intervention Images of different organs were captured with an exposure time of 2 min. The measurements of average radiance (p/s/cm2/sr) were obtained from a standard-sized ROI. Data were analyzed using the IVIS® Spectrum in Fluorescent Imager (PerkinElmer, Life Sciences, USA).

### Transmission electron microscopy

The preparation of brain slices for electron microscopy was performed as previously described. Briefly, mice were perfused with 0.9% saline followed by 4% paraformaldehyde, 1.5% glutaraldehyde in 0.1 M cacodylate buffer with 1 mM CaCl_2_. Post-fixation, the samples were dehydrated with a graded series of ethanol (30%, 50%, 70%, 80%, 90%, 95%, and 100%) and propylene oxide, and then were embedded in an epoxy resin. After the polymerization of the resin selected small sections of the CA1 region of the hippocampus were re-embedded in Durcupan blocks for sectioning. Serial 70–80 nm thick sections were collected on piliform-coated copper grids and observed using transmission electron microscopy (HT7800, HITACHI).

### Behavior tests

Forced swimming test (FST) and tail suspension test (TST) were performed to determine the antidepressant effect of HA NGs@exosomes.

#### FST

To examine the desperate behavior of mice, the experiment was conducted according to the modified version of Porsolt FST [58]. Briefly, mice were placed in a glass cylinder (12 cm in diameter) filled with 10-cm deep water (23–25℃). The mice were forced to swim for 6 min, in which the immobile time during the final 4 min was recorded, and analyzed by the ANY-maze software (Stoelting, Illinois, USA).

#### TST

ST was conducted by a computerized system. Tape was affixed to the mouse’s tail 1 cm from the tip. The mice were suspended from a horizontal bar at a height of 50 cm for 6 min. Immobile behaviors were recorded and analyzed by the ANY-maze software (Stoelting, Illinois, USA).

### Biosafety evaluation

To evaluate biosafety, blood samples were collected on the first day 1, 7th, and 30th after HA NGs@exosomes intervention. Serum ALT, TP, TBIL, BUN, CRE, and PLT levels were measured using an automatic analyzer (C8000 Roche; Hoffmann-La Roche Inc., Switzerland). Serum levels of TNF-α, IL-1β and IL-6 were measured using ELISA kits (R&D Systems, Inc.), according to the manufacturer’s instructions.

### Histological analysis

Heart, liver, spleen, lung, and kidney tissues were collected on the first day 1, 7th, and 30th after HA NGs@exosomes intervention for histological investigation using H&E staining. The specimens were fixed with 4% PFA, embedded in paraffin. A microtome was used to cut ultrathin tissue slices (4 mm). After dehydration in an alcohol gradient, sections were stained with hematoxylin and eosin. Samples were observed under a light microscope (200 ×).

### Lipid peroxidation (LPO) assay, glutathione (GSH) determination

LPO assay was carried out by determining the formation of reactive thiobarbituric acid reactive substances (TBARS) using an LPO detection kit (A106A, Jiancheng, China) as previously described [59]. Results were expressed as ng of MDA/mg of protein measured by the Lowry method. Concentrations of GSH were assayed according to a previous study with slight modification using GSH detection kit (A006-2-1, Jiancheng, China) based on the manufacturer’s instructions [60].

### Enzyme-linked immunosorbent assay (ELISA) of cytokines

TNF-α, IL-6 and IL-1β levels in medial prefrontal cortex (mPFC) and ventral hippocampus (vHPC) were quantified by sandwich ELISA method using the TNF-α ELISA kit (ZC-02567, ZhuoCai, China), IL-6 ELISA Kit (ZC-02566, ZhuoCai, China), and IL-1β ELISA Kit (ZhuoCai, China). The protocol was performed following the manufacturer’s suggestions.

### Immunofluorescence analysis

The immunofluorescence analysis for brain tissues was performed according to the previous study [61]. Mice were perfused with 4% paraformaldehyde (PFA) under anesthesia using Pelltobarbitalum Natricum. Following brain post-fixation with 4% PFA for 48 h, the brains were dehydrated by gradient ethanol, transparent with xylene, soaked in wax, and embedded. The tissue was cut into 5 μm sections via the paraffin microtome. The brain slices were baked at 60 °C for 2–8 h and deparaffinized with xylene, rehydrated with gradient alcohol, washed with PBS. After blockage with serum for 30 min at 37 °C. the samples were incubated with primary antibodies: anti-CD31(ab222783, Abcam, Cambridge, UK; 1:200 dilution); anti-Iba-1 (01919741, Wako, Osaka, Japan; 1:200 dilution), anti-PAC1 (ab183103, Abcam, Cambridge, UK; 1:200 dilution), anti-PACAP (SAB2501270, Sigma, St. Louis UK; 1:200 dilution), anti-PSD95 (sc-6926, Santa, CA, USA; 1:200 dilution), anti-MAP2 (ab32454, Abcam, Cambridge, UK; 1:200 dilution). After being washed with PBS, sections were incubated with the following secondary antibodies for 1 h at room temperature: Goat Anti-Rabbit IgG H&L (Alexa Fluor 488) (ab150077, Abcam, Cambridge, UK; 1:1000 dilution), Rabbit anti-Goat IgG H&L (Alexa Fluor 594) (A27016, Invitrogen, Paisley, UK; 1:1000 dilution), Goat Anti-Rabbit IgG H&L (Alexa Fluor 594) (ab150080, Abcam, Cambridge, UK; 1:1000 dilution), and Donkey anti-Goat IgG (H + L) (Alexa Fluor 488) (A-11,055, Invitrogen), and further counterstained for approximate 15 min with DAPI solution. After being sealed with anti-fluorescence quenching sealing solution, images of fluorescently labeled proteins were observed under a fluorescence microscope.

### Western blot

The western blot technique was applied to evaluate the expression of key proteins in PACAP/PAC1 pathway as previously described with slight modifications [62]. Briefly, protein samples (tissues were collected from mPFC and vHPC, or cells) concentrations were determined using the BCA kit (Pierce Biotechnology, Rockford, IL, USA). Then, equal amounts of protein extract were electrophoresed on a 10% polyacrylamide gel and transferred to a nitrocellulose membrane (Millipore). After blocking for 1 h at room temperature in 5% skim milk, membranes were incubated in the primary antibodies overnight at 4 ℃. The protein bands were detected with primary rabbit polyclonal antibodies: anti-PAC1(ab183103, Abcam, Cambridge, UK; 1:200 dilution), anti-PKA (4782, CST, MA, USA; 1:1000 dilution), anti-p-CREB (9196, CST MA, USA; 1:1000 dilution), anti-CREB (9197, CST, MA, USA; 1:1000 dilution), anti-BDNF (AB1534S, Millipore, St. Louis, UK; 1:1000 dilution), anti-β-Tubulin (BM1453, BOSTER, Wuhan, China; 1:10000 dilution), and secondary horseradish peroxidase (HRP)-conjugated goat anti-rabbit Ig-HRP (ab6721, Abcam, Cambridge, UK; 1:200 dilution). Blots were developed by chemiluminescence and quantitated using ImageJ software (NIH, Bethesda, MA, USA).

### Quantitative qRT-PCR

For quantitative RT-PCR analysis of *Pca1* and *Bdnf* mRNA, total RNA was collected using Trizol reagent (15,596,026, Invitrogen, USA) following the manufacturer’s instructions. qRT-PCR was performed with SYBR FAST qPCR Kit (A40425, Thermo Fisher, USA) following the manufacturer’s instructions, and performed on an ABI Prism 7000 Sequence Detection System (Applied Biosystems, USA). Relative fold changes of gene expression were calculated based on the following formula: 2^−ΔΔCt^. Primers used include the following: *Pca1*-F: TACTGTGTGTGTAACTGTGTGGG, *Pca1*-R: GCCAGCCGTAAGTAGATGCTC; *Bdnf*-F: CTCCGGGTTGGTATACTGGG, *Bdnf*-R: TCTCACCTGGTGGAACTTCTTT.

### Golgi-Cox staining

Golgi staining was performed to investigate the spine density in each group (n = 6) using Golgi-Cox OptimStain Kit (Hitobiotec, USA). Briefly, as described in the manufacturer’s instructions, the brains of mice were removed, cut into 1 cubic centimeter of tissue pieces, and placed in Golgi-Cox solution (containing Solutions 1 and 2) at room temperature for 14 days in the dark. Subsequently, brain samples were immersed in Solution 3 for 48 h at 4 ℃. After that, brain tissues were sliced into 150-µm-thick coronal slices by a vibratome (Leica CM1950, Germany). Then, the sections were placed onto gelatin-coated microscope slides and stained with solutions 4 and 5 after natural drying. Afterward, the brain slices were dehydrated with increasing concentrations of ethanol (50% ethanol for 5 min, 70% ethanol for 5 s, 95% ethanol for 5 s, and twice 100% ethanol for 5 s), then incubated in xylene for 5 min twice and coverslipped with permount. The images of spine density were captured using microscope and Leica microsystems (Leica D35578, Germany). Secondary dendritic segments of 10 mm length from 4 to 5 neurons in the CA1 area from each animal were analyzed for the number of dendritic spines.

### Electrophysiology

Long-term potentiation (LTP), another parameter for determining neural synaptic plasticity, was recorded using an *ex-vivo* brain slice as previously described [63]. Mice were anesthetized with halothane and decapitated. The brain was quickly removed and placed in artificial cerebrospinal fluid (SCF) containing the following components (in mM): 119 NaCl, 26.2 NaHCO_3_, 2.5 CaCl_2_, 2.5 KCl, 1 NaH_2_PO_4_, 1.3 MgSO_4_ and 11 glucoses (pH = 7.4). Then, 400 μm thick brain slices containing the hippocampus were taken and transferred to an interface chamber at 32 °C for 2 h of recovery prior to stimulation of the electrode and recording. A World Precision Instruments (Sarasota, FL) stimulation isolation unit and recording electrodes (filled with 2 M NaCl) were placed in the stratum radiatum (SR) layer of the CA1 region. Platinum metal microelectrodes, also placed in the SR layer, were used to evoke Field excitatory postsynaptic potentials (fEPSPs). The voltage responses were amplified using a Multiclamp 700 B amplifier and digitized using the Digidata 1322 A (Axon Instruments, Foster City, CA). After recording a stable baseline for 10 min, the input-output curves were obtained. The test stimuli were delivered at 30 s intervals. Baseline responses were monitored for at least 10 min, then three theta-burst trains (5 × 4spikes at 100 Hz) with an inter-stimulus interval of 20 s were applied, and finally, responses were acquired for 30 min post-tetanus. The Values of fEPSP amplitude were presented as mean ± SD percentage, which was normalized to the mean fEPSP slope for 10 min before weaning.

### Statistical analysis

All experiments were performed in triplicate. Data were presented as the mean ± standard deviation (SD). Statistical analyses were performed using unpaired two-tailed Student’s t-test with two-group comparisons or one-way ANOVA with multiple comparisons on GraphPad Prism software version 7.0 (GraphPad Software, San Diego, CA, USA). Statistical significance was defined as *or # *P* < 0.05, **or ## *P* < 0.01, *** or ### *P* < 0.001.

## Results and discussion

### Preparation and characterization of HA NGs@exosomes

**Fig. 1:**
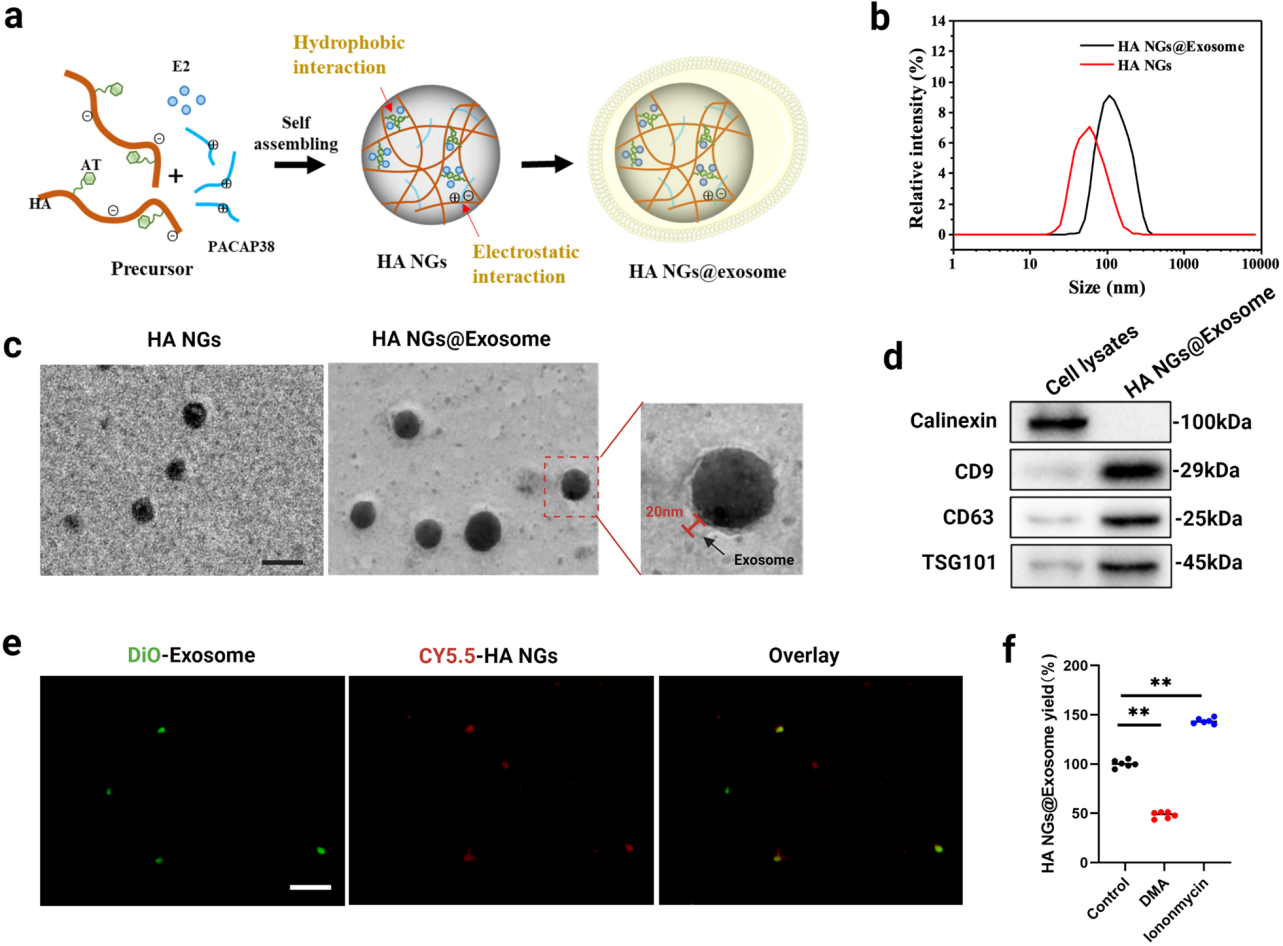
Evaluation of exosomes sheathed on HA NGs in HA NGs@exosomes. **a** Schematic illustration of the preparation of HA NGs@exosomes. Nanogels were self-assembled by HA precursors, PACAP38 peptide and estrogen were entrapped via a non-covalence bond. HA NGs fused into exosome membranes after incubating with macrophagocyte exosomes. **b** The hydrodynamic diameter of HA NGs and HA NGs@exosomes by DLS analysis. **c** TEM images of HA NGs and HA NGs@exosomes, Scale bar: 100 nm. **d** Western blotting analysis of ER marker (calnexin) and exosome markers (CD9, CD63, and TSG101) expressed in HA NGs@exosomes exocytosed from Raw264.7 cells. **e** Colocalization of DiO-Exosome, CY5.5-HA NG in HA NGs@exosomes from primary neural cells by fluorescent microscopy. Scale bar: 10 μm. **f** The yield of HA NGs@exosomes when Raw264.7 cells were pretreated with 200 µgmL^− 1^ HA NGs for 6 h and then incubated in fresh medium containing 15 nM DMA or 10 µM ionomycin for 16 h by ICP-OES. Data were represented as mean ± SD (n = 6). **P < 0.01, (one-way ANOVA)

E2 replacement therapy is widely used in treating perimenopausal depression by ameliorating the shortage of endogenous estrogen [8]. PACAP, belongs to the glucagon-secretin-vasoactive intestinal polypeptide superfamily that might contribute to the pathogenesis of certain depressive conditions [16, 64, 65]. Here, we developed a biocompatible, cell-exocytosed, and exosome-sheathed degradable nanogel to carry both PACAP and E2. As shown in Fig. 1a, the AT-modified HA (HA-AT), which serves as the backbone of NGs, was co-assembled with E2 and PACAP38 to form NGs via hydrophobic and electrostatic interactions, respectively. Then, exosome-sheathed NGs (HA NGs@exosomes) were prepared and identified by the co-incubation method. Specifically, the HA NGs obtained above were dispersed in an aqueous solution containing exosomes extracted from Raw264.7 cells [66], ultra-sounded for 20 min, and then incubated for 30 min at 37℃. DLS revealed that HA NGs@exosomes have an obvious increase in size compared with HA NGs (Fig. 1b). Then, the morphology of HA NGs@exosomes was characterized by TEM images. NG showed a relatively monodisperse size distribution of around 80 nm. HA NGs@exosomes displayed near-spherical morphology, and ca. 20 nm thick membrane appeared on the surface of HA NGs@exosomes (Fig. 1c). To further confirmed that HA NGs was sheathed with exosomes, exosome biomarkers CD9, CD63, and TSG101 were also detected in Fig. 1d, calnexin, an endoplasmic reticulum (ER) marker, was only detected in whole cell lysates, not in HA NGs@exosomes. The characterization of purified exosomes was shown in Additional file 1: Figs. S1a, S1b and S1c. The dissociation process of HA NGs@exosomes was detected by TEM (Additional file 1: Fig. S1d). Then, exosomes and HA NGs were labeled with 3,3′-dioctadecyloxacarbocyanine perchlorate (DiO) and Cyanine5.5 (CY5.5) dyes, respectively. As shown in Fig. 1e, the green fluorescence of DiO-exosomes and red fluorescence of CY5.5-HA NGs were overlaid, which is direct proof of the formulation of HA NGs@exosomes. Moreover, we found that the yield of HA NGs@exosomes can be greatly influenced either by exosome release inhibitors, dimethyl amiloride (DMA) [67], or by exosome release promoter, ionomycin [68] (Fig. 1f).

**Fig. 2:**
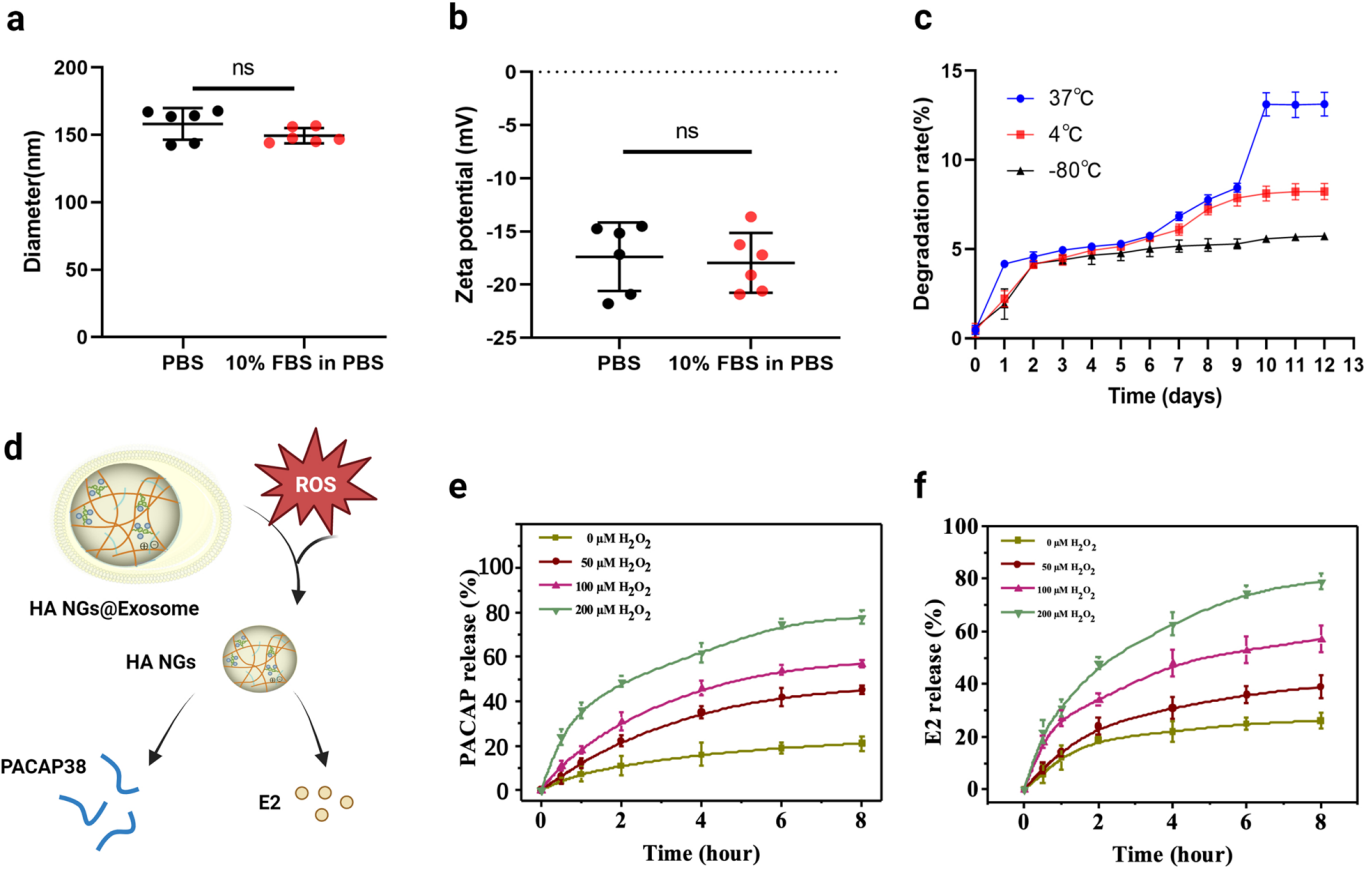
Characterization of HA NGs@exosomes. **a** The hydrodynamic diameter of HA NGs@exosomes incubating in PBS with or without 10% FBS. **b** Zeta potential of HA NGs@exosomes incubating in PBS with or without 10% FBS. **c** Degradation behavior of HA NGs@exosomes in PBS at 37 °C, 4 °C, and − 80 °C for different time intervals. **d** Schematic illustration of ROS-responsive ability of HA NGs@exosomes. The release profile of PACAP (**e**) and E2 (**f**) in HA NGs@exosomes in the presence of an increasing concentration of H_2_O_2_ solution, was determined using UV-vis spectrophotometer and HPLC analysis. Data were presented as mean ± SD (n = 6). *ns* not significant, (one-way ANOVA)

As mentioned previously, HA NGs@exosomes as drug carriers were loaded with PACAP and E2. The successful loading of PACAP significantly improved the zeta potential of NGs compared with that of pure HA NGs (Additional file 1: Fig. S2). As shown in Table 1, the EE for PACAP and E2 were 70.81% and 48.12%, respectively, while the calculated LC for PACAP and E2 were 11.7% and 16%, respectively. PACAP and E2 loading did not significantly change the diameter and zeta potential of HA NGs@exosomes, they remained almost constant even after incubating in PBS with or without 10% FBS for 6 days (Fig. 2a and b). Furthermore, relatively little degradation of HA NGs@exosomes was detected at different temperatures before 7 days, indicating relatively stable properties of HA NGs@exosomes (Fig. 2c). It is worth emphasizing that the HA NGs@exosomes exhibited ROS-responsive release (Fig. 2d). As shown in Fig. 2e and f, the release ratio of E2 and PACAP increased significantly with the elevation of H_2_O_2_ concentration, which is probably due to the oxidative degradation of HA [69]. This triggered release of the guest molecules is of paramount importance in avoiding damage to normal tissue caused by drug system distribution, thereby enhancing the targeted site release.

To conclude, these results indicated that, in contrast to naked HA NGs, HA NGs@exosomes display a distinguished core-shell structure with relatively stable properties. Moreover, HA NGs@exosomes have great ROS-responsive ability.

**Table 1:** Loading efficiency of PACAP and E2

Drug loaded	Drug loading	Encapsulation Efficiency
PACAP	11.7%	70.18±1.5%
E2	16%	48.12±1.1%

### Cellular uptake and BBB penetration of HA NGs@exosomes

**Fig. 3:**
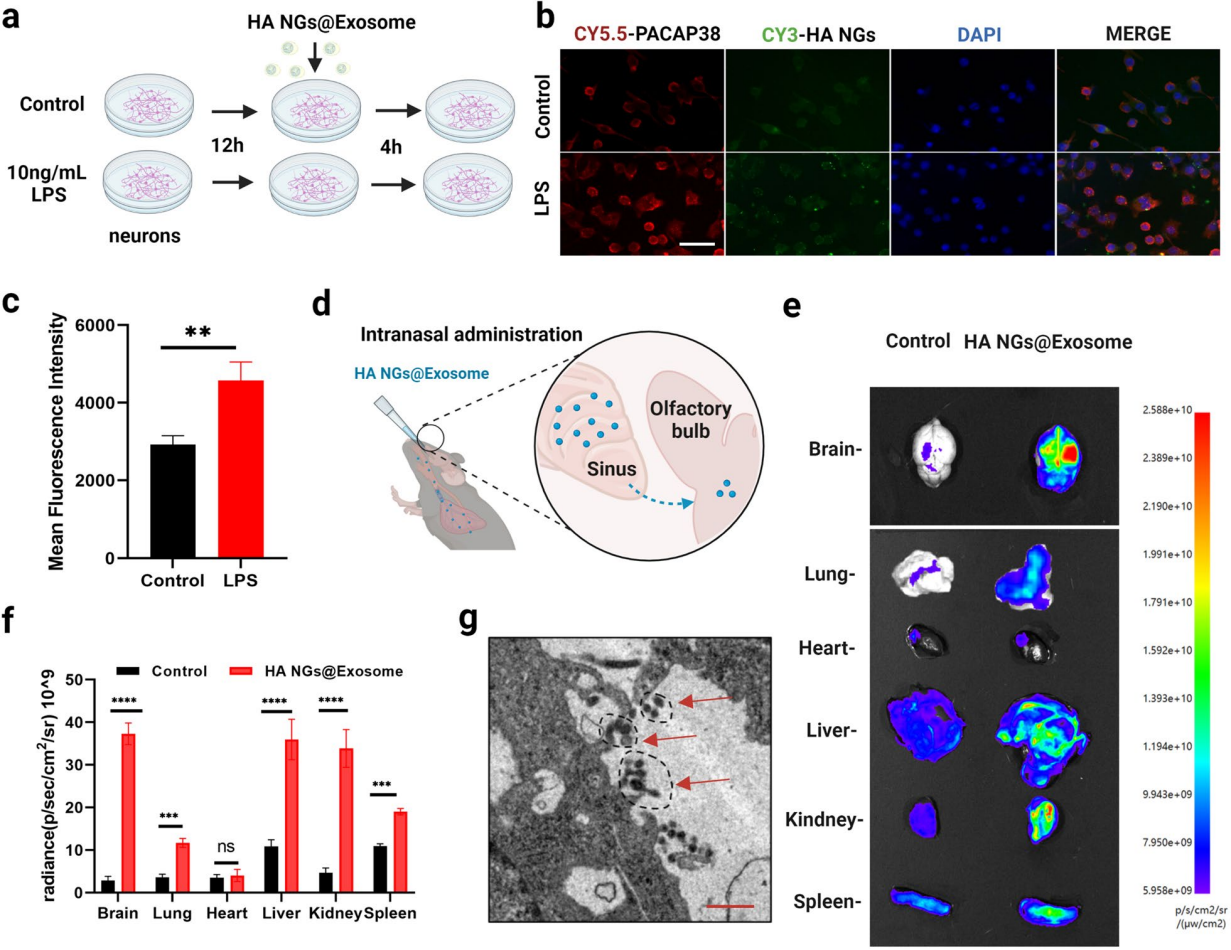
Cellular uptake and accumulation of HA NGs@exosomes in vitro and in vivo. **a** The schematic illustration of the in vitro experiment. Primary neurons were treated with 10ng/ml LPS and incubated for 12 h. Afterward, HA NGs@exosomes were added to each plate and co-incubated for 4 h. **b** Colocalization of CY5.5-PACAP, CY3-HA NGs in HA NGs@exosomes from primary neuronal cells by fluorescent microscopy. Scale bar: 50 μm. **c** Quantification of mean fluorescence intensity in the control or LPS group. **d** The intranasal delivery route of HA NGs@exosomes administration. HA NGs@exosomes across the nasal epithelium into the brain. **e** The in vivo distribution of labeled HA NGs@exosomes in different organs was visualized. **f** The particles distributed in each organ was determined based on the fluorescent intensity. **g** Localization of HA NGs@exosomes at a sub-cellular level by TEM. Dashed circles indicated the accumulated HA NGs@exosomes. Scale bar: 2 μm. Data were presented as mean ± SD for three independent assays. **P < 0.01, ***P < 0.001, ****P < 0.0001, *ns* not significant, (Student's, t-tests)

To investigate whether HA NGs@exosomes possess efficient cellular uptake in the different cellular states in vitro, primary neuronal cells were stimulated with 10ng/ml LPS and then incubated with HA NGs@exosomes (CY5.5-labeled PACAP and CY3-labeled HA nanogels) for 4 h (Fig. 3a). Immunofluorescence staining showed the co-localization of PACAP and HA nanogels, indicating the successful encapsulation of PACAP into HA NGs@exosomes in primary neuronal cells (Fig. 3b). Moreover, LPS stimulated model had a better performance of cellular uptake than that of the control group, probably because of the opening of the BBB under the LPS-stimulated inflammatory state (Fig. 3c). To better evaluate the cellular uptake of HA NGs@exosomes in vivo, C57BL/6 mice were intranasally administrated with radio-labeled HA NGs@exosomes (Fig. 3d). The fluorescence imaging 24 h after the intervention revealed that, even in healthy mice, the HA NGs@exosomes can be accumulated in various organs, including the brain, lung, liver, kidney, and spleen. Furthermore, as shown in Fig. 3e, a relative high level of DiR-HA NGs@exosomes traveled to brain, liver, kidney, spleen and lung compared to free DiR dye (Fig. 3e). The fluorescence intensity of these organs in the HA NGs@exosomes group was significantly higher than that of the control group. It was obvious that the HA NGs@exosomes treated mice displayed significant stronger fluorescence than free dye treated mice in brain, lung, liver, kidney and spleen. In brain, the fluorescence intensity in the HA NGs@exosomes group was almost 13 times stronger than the control. (Fig. 3f). As shown in Additional file 1: Fig. S3, HA NGs@exosomes has the ability to cross the BBB, but not HA NGs. Additionally, TEM was induced to observe the localization of HA NGs@exosomes at a sub-cellular level. In Fig. 3g, small circles with dashed lines indicated the existence of HA NGs@exosomes in the cytoplasm of neuronal cells in CA1 region of the hippocampus.

**Fig. 4:**
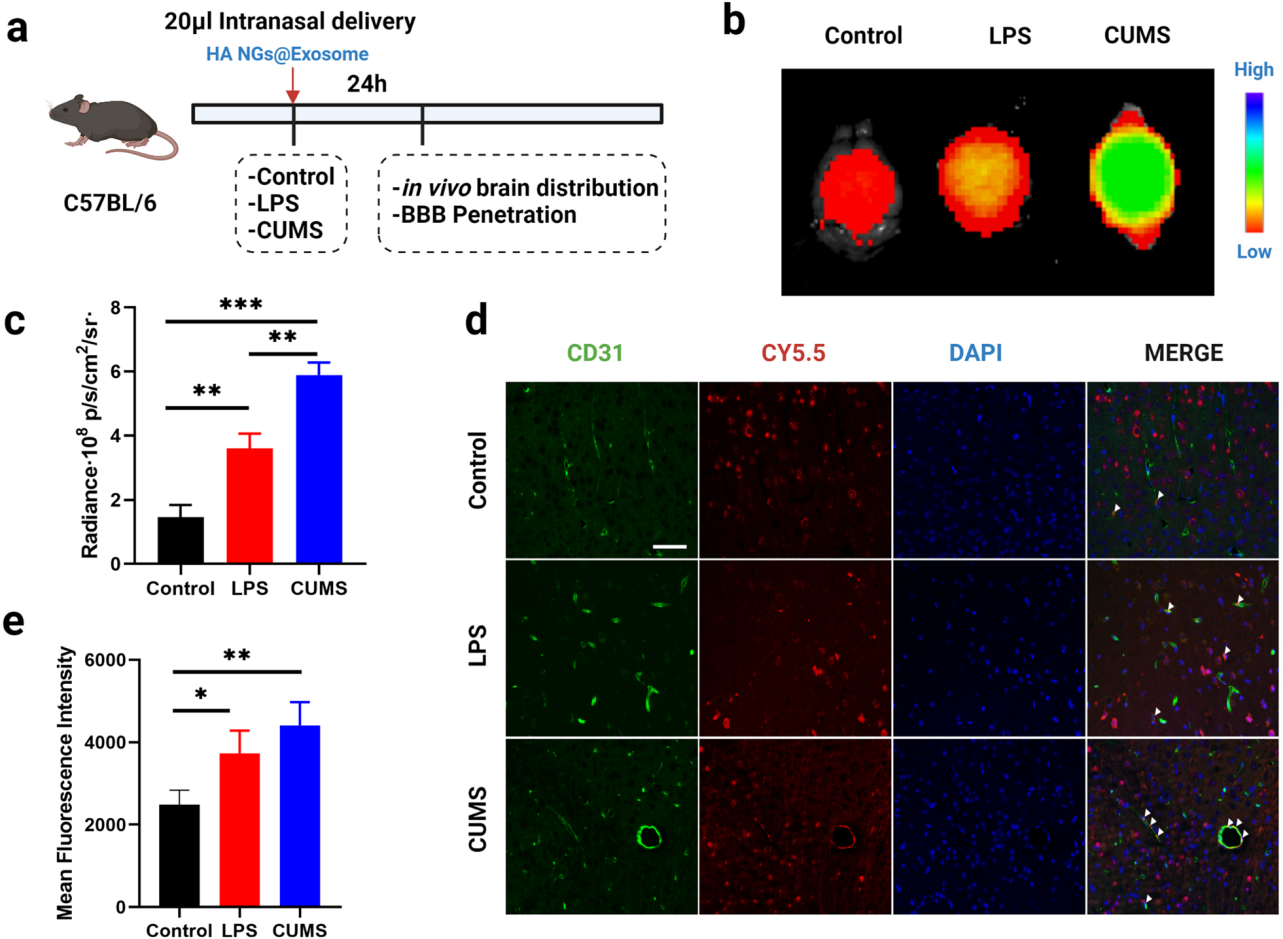
The brain distribution and BBB penetration of HA NGs@exosomes under pathological conditions. **a** The schematic illustration of the experiment. Healthy control, LPS-treated and CUMS mice were intranasally administrated with HA NGs@exosomes, 24 h later, related parameters were detected. **b** Brain distribution of HA NGs@exosomes in healthy mice, LPS-treated mice, or CUMS-induced mice was determined by IVIS® Spectrum in Fluorescent Imager. **c** Quantification of average radiance (p/s/cm^2^/sr) in Control, LPS-treated mice, or CUMS-induced mice. **d** Colocalization of CD31-Endothelium and CY5.5-HA NGs@exosomes were detected by fluorescent microscopy. Scale bar: 50 μm. **e** Quantification of mean fluorescent intensity in each group. Data were presented as mean ± SD for three independent assays. *P < 0.05, **P < 0.01, ***P < 0.001, (one-way ANOVA)

Further, healthy mice, LPS-treated mice, or CUMS-induced mice were also intranasally administrated with radio-labeled HA NGs@exosomes to determine the in vivo accumulation of HA NGs@exosomes under pathological conditions. After the intervention for 24 h, whole-brain scanning was carried out to obtain static fluorescence images (Fig. 4a). Higher fluorescence intensity can be seen in CUMS mice, compared to both the control group and LPS group (Fig. 4b). Moreover, with measurements of average radiance (p/s/cm^2^/sr), CUMS model reached around 6 radiance·10^8^, which was significantly higher than that in LPS-treated mice or healthy mice, indicating a satisfactory accumulation of HA nanogels in the CUMS model (Fig. 4c). In addition, immunofluorescence revealed that the HA NGs@exosomes in CUMS group had a better BBB penetration performance (but not significant), as compared to that in the LPS group (Fig. 4d and e). These results demonstrate that HA NGs@exosomes display strong cellular uptake and efficient accumulation not only in primary neuronal cells but also in the LPS and CUMS-induced mice model. Moreover, HA NGs@exosomes can penetrate BBB more effectively in the CUMS model.

### Rapid-onset and sustained antidepressant effect of HA NGs@exosomes without side effects

**Fig. 5:**
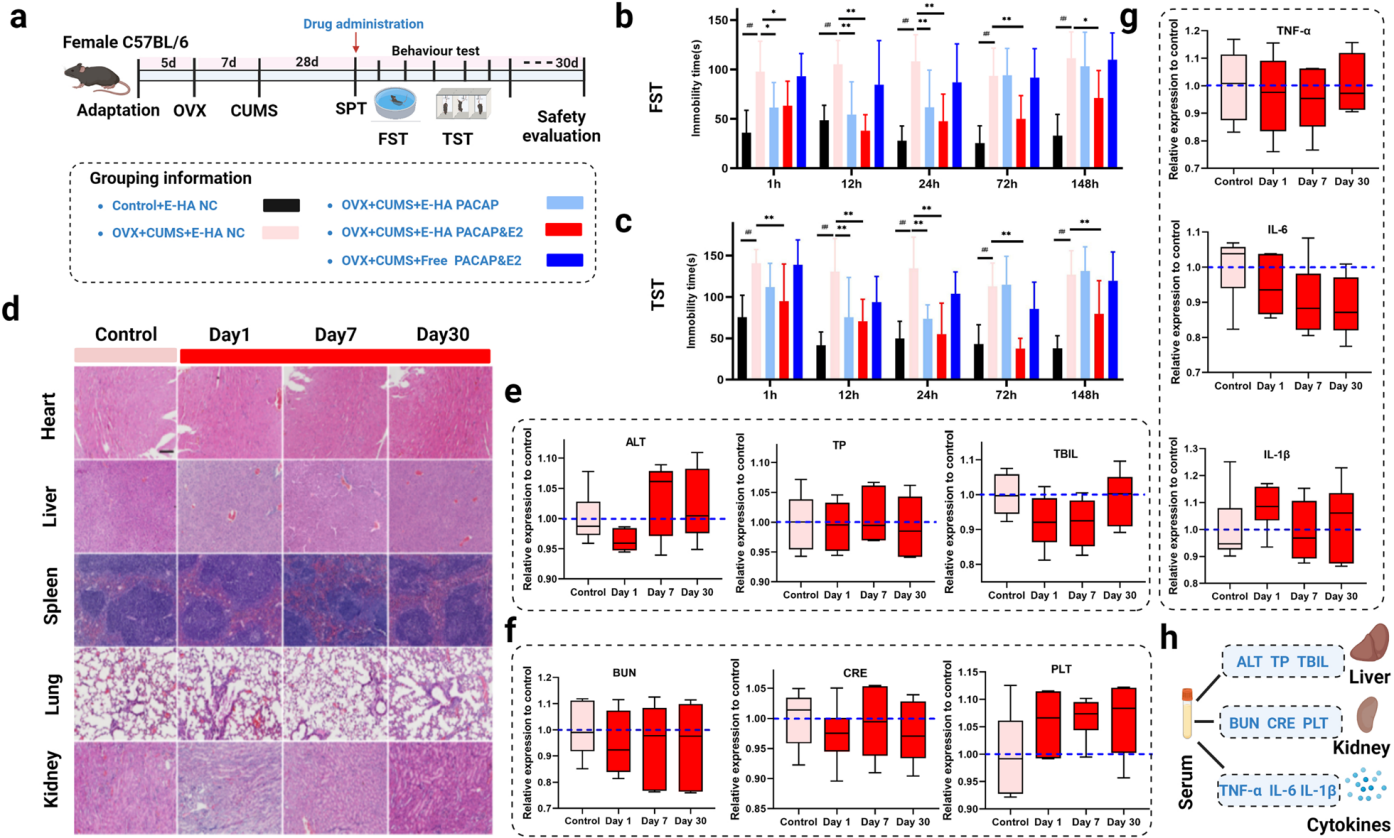
The effect of HA NGs@exosomes on depressive-like behaviors and its safety evaluations. **a** Timeline of the experimental series investigating the anti-depressant effect of HA NGs@exosomes and its safety. Grouping information was listed. The immobility time of mice (n = 10 in each group) in FST (**b**) and TST (**c**) was tested at various time points (1 h, 12 h, 24 h, 72 h, 148 h) following treatment with free PACAP&E2, E-HA PACAP, E-PACAP&E2 or their blank control vehicles (E-HA NC). **d** Histological investigations of the heart, liver, spleen, lung, and kidney were conducted using H&E staining on the 1st day, 7th day, and 30th day after HA NGs@exosomes intervention. Scale bar: 100 μm. The enzyme activities of the liver (ALT, TP, TBIL) (**e**); Renal function parameters (BUN, CRE, PLT) (**f**); and cytokines in serum (TNF-α, IL-6, IL-1β) (**g**) were tested on the first day, 7th day, and 30th day after HA NGs@exosomes intervention (n = 6). **h** The summary of the safety evaluation of parameters in the liver, kidney, and serum. Data were presented as mean ± SD, #*P* < 0.05, ##*P* < 0.01 vs. Control + E-HA NC; *P < 0.05, **P < 0.01 vs. OVX + CUMS + E-HA NC, (two-way ANOVA with Bonferroni’s multiple comparisons test for **b**, **c** and one-way ANOVA for **e**, **f** and **g**)

Given that HA NGs@exosomes exhibited efficient cellular uptake and BBB penetration especially in CUMS-induced mice model, the antidepressant effect of HA NGs@exosomes was further explored. We selected ovariectomy combined with CUMS (OVX-CUMS) model to mimic a perimenopausal depressive state. Figure 5a showed the schematic diagrams of the time points of drug intervention, behavior tests, and safety evaluation. The SPT was used to determine whether the models were established successfully (Additional file 1: Fig. S4a). Then, HA NGs@exosomes encapsulated with PACAP&Estrogen (E-HA PACAP&E2), HA NGs@exosomes encapsulated with PACAP only (E-HA PACAP), Free PACAP&E2, or their blank control vehicles (E-HA NC) were intranasally administrated to OVX-CUMS induced female mice at a dosage of 20 ul. Afterward, classical depressive-like behaviors tests, FST, and TST were performed to identify the antidepressant effect of HA NGs@exosomes in OVX-CUMS model. TST and TST were performed at various time points (1 h, 12 h, 24 h, 72 h, 148 h) following drug intervention. As shown in Fig. 5b and c, the OVX-CUMS group showed a dynamic increased immobility time in all groups compared with the control group without CUMS. Treatment with E-HA PACAP and E-PACAP&E2 significantly decreased the immobility time as early as 1 h after the initial intervention and lasted until 24 h. However, the effect of E-HA PACAP gradually diminished at later time points, whereas E-PACAP &E2 revealed a sustained antidepressant effect throughout the experiment. Free PACAP&E2 showed no antidepressant effect at all time points. In addition, uterine atrophy can be observed in OVX-CUMS group, which recovered significantly after E-PACAP&E2 treatment (Additional file 1: Fig. S4b). Subsequently, we addressed several experiments regarding the safety of HA NGs@exosomes. H&E staining on the heart, liver, spleen, lung, and kidney was performed. No morphological or histopathological damage appeared in these organs after HA NGs@exosomes intervention for up to 30 days (Fig. 5d).

In addition, enzyme activities reflecting hepatocyte damage such as alanine aminotransferase (ALT), total protein (TP), and total bilirubin (TBIL); parameters reflecting renal function such as blood urea nitrogen (BUN), serum creatinine (CRE), platelet (PLT); as well as plasma cytokines levels including TNF-α, IL-6, IL-1β were tested at the1st, 7th and 30th day, respectively. (Figure 5e and f g). Results showed that these factors were not significantly altered in the HA NGs@exosomes-treated group compared with the controls, suggesting the safe antidepressant effect of HA NGs@exosomes encapsulated with PACAP&E2. The summary schematic diagrams were shown in Fig. 5h.

### Alteration of ROS-induced peroxidation and microglial state by HA NGs@exosomes

**Fig. 6:**
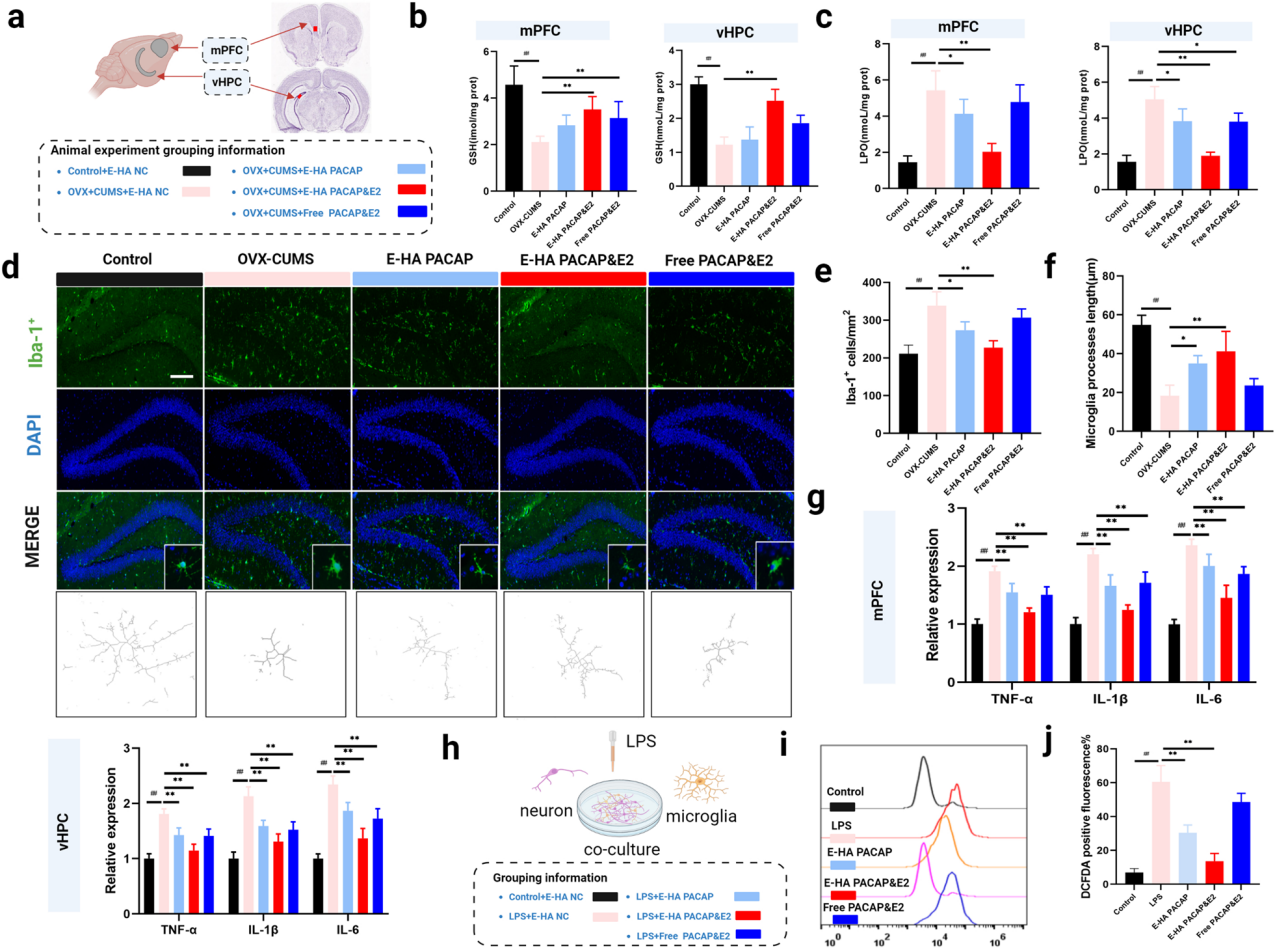
The effect of HA NGs@exosomes on oxidative stress and microglial activation. **a** The location of the mPFC and vHPC of the mice brain. The levels of GSH (**b**) and LPO (**c**) in the mPFC and vHPC were tested in different groups (n = 6 for each group). **d** Morphology and numbers of microglia were observed with immunofluorescence staining. Scale bar: 100 μm. The number of Iba-1^+^ cells **e** and microglial processes length (**f**) were quantified. **g** The levels of cytokines including TNF-α, IL-1β, and IL-6 were tested in the mPFC and vHPC regions in each group (n = 6 for each group). **h** The schematic illustration of the neuron-microglia co-culture model, as well as the grouping information. **i** The spectrum of ROS generation in each group. **j** The intracellular ROS generation in each group was qualified. Data were presented as mean ± SD. ##*P* < 0.01 vs. Control + E-HA NC; *P < 0.05, **P < 0.01 vs. OVX + CUMS + E-HA NC. (two-way ANOVA with Bonferroni’s multiple comparisons test for G, and one-way ANOVA for **b**, **c**, **e**, **f**, and **j**)

Besides efficient cellular uptake and rapid-onset antidepressant effect, how HA NGs@exosomes exert their function should be further explored. Consequently, a series of experiments were carried out in distinct cerebral areas, specifically, the mPFC and vHPC, as these regions are predominantly implicated in the pathophysiology of depressive disorders [70]. Repeatedly, free PACAP&E2, E-HA PACAP, E-PACAP&E2, and E-HA NC were intranasally given to OVX-CUMS induced female mice. GSH and LPO levels were investigated at the end-point of treatment (Fig. 6a), as well as the level of ROS (Additional file 1: Fig. S5). As a result, the GSH level was dramatically decreased in the OVX-CUMS model in both mPFC and vHPC regions, while with the treatment of E-PACAP&E2, GSH level increased obviously (Fig. 6b). In contrast to the rise of GSH, intervention with E-PACAP&E2 or E-HA PACAP significantly decreased the level of LPO in both regions, moreover, E-PACAP&E2 had a stronger effect (Fig. 6c). In addition, as shown in Additional file 1: Fig. S5, the level of ROS was significantly increased in both regions, and treatment with E-HA PACAP and E-HA PACAP&E2 markedly decreased the elevated level of ROS. These data indicated that in OVX-CUMS model, ROS-induced peroxidation can be greatly prohibited by HA NGs@exosomes, especially with encapsulated PACAP&E2. Additionally, microglial activation was observed with immunofluorescence staining in the hippocampus (Fig. 6d). The number of Iba-1^+^ cells was significantly increased and microglial process length was shortened in OVX-CUMS model, indicating an inflammatory state in the local brain. However, with the intervention of HA NGs@exosomes, the number of Iba-1^+^ cells were reduced, especially in the E-PACAP&E2 group, while microglial process length was surely recovered (Fig. 6e and f). Furthermore, cytokines, e.g., TNF-α, IL-1β and IL-6 were determined in both mPFC and vHPC regions. Results indicated that the level of these factors was significantly up-regulated in the OVX-CUMS-induced model. However, with the treatment of HA NGs@exosomes containing PACAP or PACAP&E2, even treated with free PACAP&E2, the levels of these cytokines were markedly decreased (Fig. 6g). We further confirmed the anti-inflammatory effect of HA NGs@exosomes in the neuron-microglia co-culture model. After LPS stimulation, cells were treated with free PACAP&E2, E-HA PACAP, E-PACAP&E2, and E-HA NC (Fig. 6h). In response to LPS, the generation of ROS was significantly increased, whereas, treatment with E-HA PACAP or E-PACAP&E2 significantly inhibited the level of ROS, suggesting the excellent antioxidant effect of HA NGs@exosomes containing PACAP&E2 or PACAP. No antioxidant effect can be detected in the free PACAP&E2 group (Fig. 6i and j). The cytokines profile in each group was provided in Additional file 1: Figure S6a, b and c.

Overall, these data demonstrated that ROS-induced peroxidation and inflammatory conditions can be ameliorated by HA NGs@exosomes containing PACAP or PACAP&E2, in which PACAP&E2 had a better effect. Free PACAP&E2, however, was not as effective as E-PACAP&E2 in reducing cytokines and oxidative stress.

### Improvement of PACAP/PAC1 pathway-mediated neural synaptic plasticity by HA NGs@exosomes

**Fig. 7:**
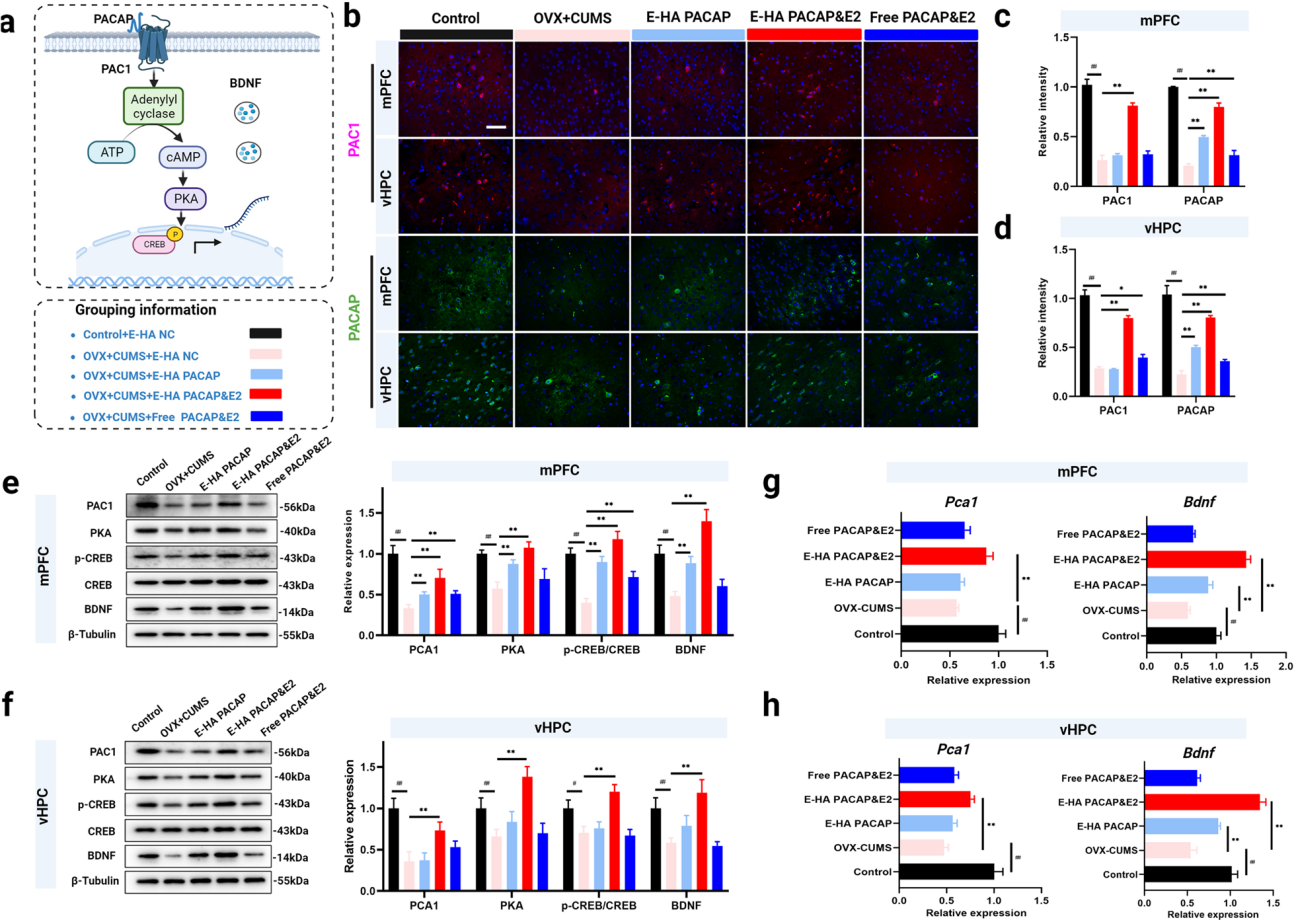
The effect of HA NGs@exosomes on key proteins involved in the PACAP/PAC1 pathway. **a** Illustration of the key factors involved in the PACAP/PAC1 pathway, as well as the grouping information. **b** Expression of PAC1 and PACAP was detected by immunofluorescence staining, scale bar: 50 μm. The relative intensity of PAC1 and PACAP were quantified in the mPFC region (**c**) and vHPC region (**d**). The expression of PAC1, PKA, p-CREB, CREB, and BDNF were determined by western blot and further quantified in the mPFC region (**e**) and vHPC region (**f**). *Pca1* and *Bdnf* mRNA was determined in the mPFC region (**g**) and vHPC region (**h**). Data were presented as mean ± SD (n = 6 for each group). ##*P* < 0.01 vs. Control + E-HA NC; *P < 0.05, **P < 0.01 vs. OVX + CUMS + E-HA NC, (two-way ANOVA with Bonferroni’s multiple comparisons test for **c**, **d**, **e**, **f**, and one-way ANOVA for **g**, and **h**)

To further understand the therapeutic mechanism of HA NGs@exosomes in treating perimenopausal depression, several key factors (Fig. 7a) involved in the pathological change of depression were investigated. The expression of PAC1 and PACAP was shown with immunofluorescence staining in mPFC and vHPC (Fig. 7b). As a quantification, in the mPFC region, treatment with E-PACAP&E2 could reverse the reduction of PACAP and PACAP type I receptor (PAC1) caused by OVX-CUMS, while E-HA PACAP or free PACAP&E2 appeared to not affect the expression of PAC1 (Fig. 7c). Similar results were obtained in the vHPC region (Fig. 7d). Furthermore, the expression of key factors of the PACAP/PAC1 signaling pathway involving PAC1, PKA, p-CREB, CREB, and BDNF were clarified by western blot in both animal and LPS-induced neuroinflammatory challenges models (Fig. 7e and f, Additional file 1: Fig. S7). In both mPFC and vHPC regions, the level of PAC1, PKA, p-CREB/CREB, and BDNF was dramatically up-regulated in the E-PACAP&E2 group which reversed the reduction of these factors in the OVX-CUMS group. Likewise, E-PACAP treatment showed a profound effect on the up-regulation of PAC1, PKA, p-CREB/CREB, and BDNF in the mPFC region (Fig. 7e and f). Interestingly, E-HA PACAP alone can increase the expression of key factors of the PACAP/PAC1 pathway in mPFC while having no effect in vHPC region. More work has been done to determine the expression of PCA1 and BDNF at the transcription level by qRT-PCR. Results demonstrated that expression of *Pca1* mRNA and *Bdnf* mRNA was up-regulated under the treatment of E-PACAP&E2 in both regions, while free PACAP&E2 seems to have no effect affecting gene expression of PCA1 and BDNF (Fig. 7g and h). Moreover, the expression of *Bdnf* mRNA but not *Pca1* mRNA can be also affected by E-HA PACAP. These results strongly demonstrated the superior and multifaceted therapeutic efficacy of E-PACAP&E2 in OVX-CUMS model, suggesting that exosome-encapsulated PACAP&E2 have a much more profound effect than free drugs. Moreover, HA NGs@exosomes might exert an anti-depressant effect by attenuating oxidative and inflammatory states, meanwhile affecting the expression of key factors of the PACAP/PAC1 pathway.

**Fig. 8:**
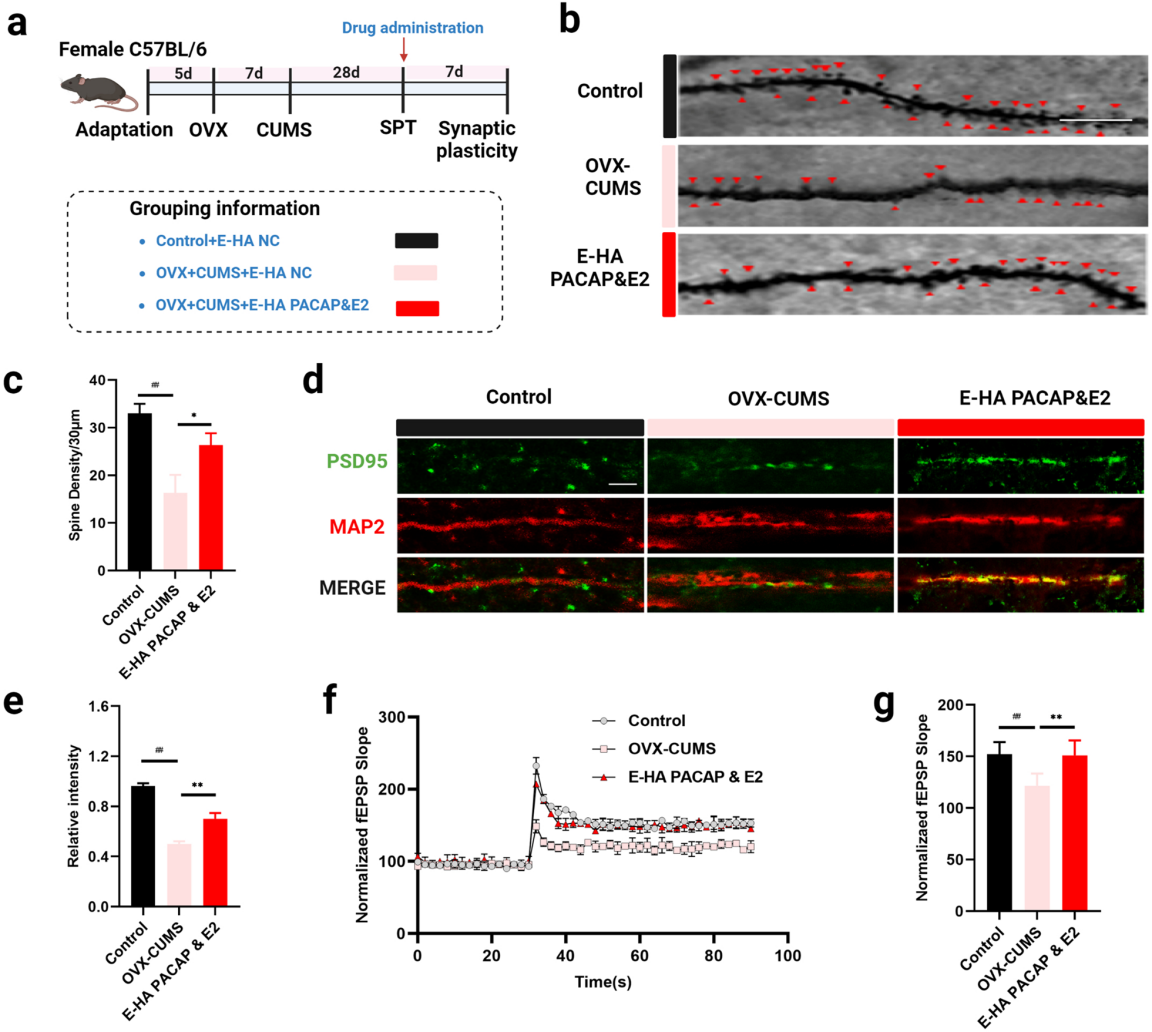
The effect of HA NGs@exosomes on neural synaptic plasticity. **a** Timeline and grouping information for experimental series investigating synaptic plasticity. **b** The dendritic spines density in Control, OVX-CUMS, and E-HA PACAP&E2 group was observed with Golgi-Cox staining. A small red triangle indicated spines. Scale bar: 10 μm. **c** Spine density in each group was quantified. Data were presented as mean ± SD (n = 3). **d** Immunofluorescence staining of PSD95, and MAP2 was shown in each group. Scale bar: 10 μm. **e** The relative intensity of PSD95 was quantified. **f** fEPSP-evoked synaptically in dendritic tuft by direct electrical stimulation was shown for 90 s. **g** Normalized fEPSP was quantified in each group. Data were presented as mean ± SD (n = 6). ##*P* < 0.01 vs. Control + E-HA NC; *P < 0.05, **P < 0.01 vs. OVX + CUMS + E-HA NC, (one-way ANOVA test)

Given that HA NGs@exosomes containing PACAP&E2 but not free drugs had a satisfactory effect on the alteration of cellular state and key factors of PACAP/PAC1 pathway. We further explored the role of E-HA PACAP&E2 in neuronal plasticity (Fig. 8a). Dendritic spines were indicated with small red triangles in different groups (Fig. 8b). It was shown that treatment with E-PACAP&E2 could compromise the reduction of spines caused by OVX-CUMS, suggesting that E-PACAP&E2 could promote neural synaptic plasticity in depressed conditions (Fig. 8c). Furthermore, immunofluorescence staining of postsynaptic density protein-95 (PSD95) and MAP2 in neural dendrites was shown (Fig. 8d). E-PACAP&E2 significantly increased the fluorescence intensity of PSD95 in OVX-CUMS mice model (Fig. 8e). Additionally, PSD95 was also determined in the neuroinflammatory challenges model with stimulation of LPS (Additional file 1: Fig. S8). Correspondingly, *ex-vivo* recording of field excitatory postsynaptic potential (fEPSP) in the CA1 of the hippocampus was performed to verify synaptic plasticity (Fig. 8f). Treatment with E-PACAP&E2 markedly enhanced the OVX-CUMS-induced fEPSP in the hippocampal CA1 pyramidal neurons, indicating a negative regulation of E-PACAP&E2 in the development of long-term potentiation (LTP) of the hippocampus in OVX-CUMS mice (Fig. 8g).

## Discussion based on the previous results

The targeted delivery of therapeutic agents to the CNS through nanoparticle-based drug delivery systems has provided significant benefits in the treatment of CNS diseases [71, 72]. Generally, BBB acts as a protective structure for preventing bloodborne or exogenous pathogens, meanwhile, maintaining the homeostasis of the microenvironment of the brain [73]. However, like the two sides of a coin, BBB would restrict the pharmacological efficacy of various drugs and present formidable challenges in developing CNS drug delivery systems. Over the past few decades, various drug delivery strategies have been developed to transport drug molecules across the BBB [74]. Among them, nanoscale drug carriers are demonstrated as an emerging transport technique for drug delivery without disrupting the structure and function of BBB [75, 76]. The size of nanoparticles is usually less than 100 nm which is an ideal property across the BBB. Besides, satisfied biodegradable and biocompatible features, prolonged blood circulation time, and non-immunogenic characteristics are also crucial to brain drug delivery [77].

Using natural biomaterials to coat nanoparticles provides a promising means to achieve efficient accumulation in target cells, and the same time, reduce the immune clearance in complex in vivo environments [78, 79]. Nowadays, several natural vesicles such as red cell membranes, liposomes, or exosomes-coated nanoparticles have been widely used as carriers for ideal pharmaceutical applications of the brain [80–83]. Among them, exosomes, sub-micrometer-sized vesicles, have attracted much attention. Exosomes are 40 to 200 nm membrane-encased vesicles secreted by cells and formed with the cell plasma membranes [37, 84]. Because these vesicles are shed by cells, they have certain unique advantages such as small size, non-immunogenicity, excellent biodegradability, and biocompatibility. In the present study, we created exosome-sheathed HA NGs to load PACAP and E2 for efficient neural targeting. Moreover, the increase of H_2_O_2_ concentration triggered the degradation of NGs, thereby facilitating the release of PACAP and E2 to enhance the pharmacological properties of these drugs based on oxidative stimulus level.

Conventional pharmacological therapy for depression is the oral administration of antidepressant drugs [85]. Perimenopausal depression, however, requires additional E2 replacement [6]. The side effects of these agents call for optimized routes of administration to reliably bypass the BBB and directly target the brain. Intranasal administration has been highlighted as the only non-invasive route of brain drug delivery due to the unique connection between the Olfactory Bulb and the brain [86]. In our study, HA NGs@exosomes were intranasally administrated to the perimenopausal depressive female mice model. Surprisingly, the rapid antidepressant effect of HA NGs@exosomes could be observed as early as 1 h after the initial intervention, suggesting that nasal administration of HA NGs@exosomes has a more rapid effect than conventional oral antidepressants, e.g., selective serotonin reuptake inhibitor (SSRIs). Moreover, HA NGs@exosomes produced no morphological or histopathological damage to major organs, and did not affect certain liver enzyme activities (ALT, TP, TBIL), indicating the safety of HA NGs@exosomes. Furthermore, upon traversing the BBB and entering the brain parenchyma, the membrane of HA NGs@exosomes could potentially be damaged by ROS. This could result in the release of drugs, which have the ability to reduce oxidative stress while concurrently diminishing the secretion of cytokines such as TNF-α, IL-6, and IL-1β released by microglia.

PACAP modulates various physiological processes via PACAP receptor type 1 (PAC1R) or vasoactive intestinal peptide/PACAP receptor [87]. Interestingly, PACAP/PAC1 receptor pathway showed a sex-specific response in post-traumatic stress disorder [18], suggesting that estrogen may affect PACAP/PAC1 system. The previous study has also shown that PACAP deficiency in mice results in increased depression-like behavior [64], indicating an important role of PACAP in the pathogenetic development of depression. Taking these results into consideration, our study aimed to explore whether the combination of PACAP and E2 would have a cooperative effect on the treatment of perimenopausal depression.

**Fig. 9:**
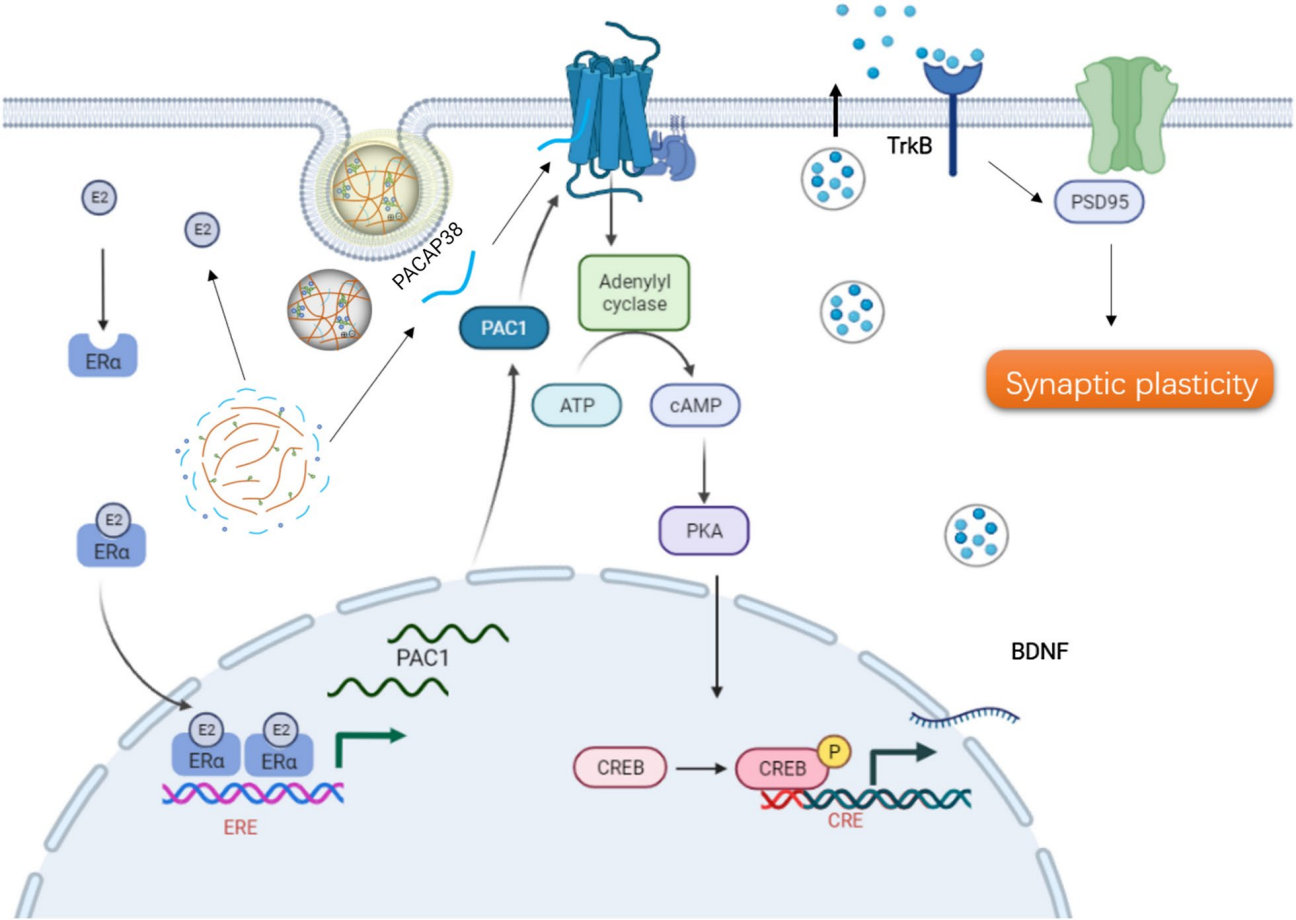
The possible mechanisms of how HA NGs@exosomes promote synaptic plasticity in neurons through the PACAP/PAC1 pathway. After uptake by neuronal cells, PACAP and E2 can be released from HA NGs@exosomes. E2 binds with ERα and promoted the expression of PAC1. In parallel, PACAP activated PACAP/PAC1 pathway and finally enhanced the expression of BDNF. BDNF binds with its receptor TrkB and promotes the expression of PSD95. ERα, estrogen receptor; ERE, estrogen response element; TrkB, tropomyosin receptor kinase B

With this point in mind, several series of experiments were performed on different brain regions. Our findings suggested that PACAP and E2 together (i.e., exosomes sheathed with PACAP and Estrogen, named HA NGs@exosomes) have more profound therapeutic efficacy in OVX-CUMS model than single or free drugs. Moreover, HA NGs@exosomes have multiple functions including (1) attenuating oxidative and inflammatory conditions; (2) regulating the expression of key factors of the PACAP/PAC1 pathway, e.g., PAC1, PKA, CREB, and BDNF. (3) improving synaptic plasticity. These effects are very likely mediated by a combination of PACAP and E2, more important, their cooperative enhancement effect since there is evidence that the PACAP/PAC1 system’s interaction with E2 in the response to chronic stress in females: E2 can increase the expression of *ADCYAP1R1* mRNA (i.e., *ADCYAP1R1* encode PAC1) through ligand activation of estrogen receptors alpha and binding to an estrogen response element within the gene [18, 88]. The possible mechanisms were presented in Fig. 9. However, more potential interaction mechanisms between PACAP/PAC1 system and E2 need to be further clarified.

## Conclusion

In summary, the current study successfully developed exosome-sheathed nanogels that are responsive to ROS and loaded with PACAP and Estrogen for the treatment of perimenopausal depression. These hybrid nanogels, referred to as HA NGs@exosomes, were found to have efficient cellular uptake in vivo. Intranasal intervention with HA NGs@exosomes in female ovariectomy CUMS-induced mice showed a significant improvement in depressive-behavior tests. Moreover, it was observed that HA NGs@exosomes could potentially exert their therapeutic effect through several mechanisms, including the reduction of proinflammatory cytokine levels, promotion of neural synaptic plasticity, and regulation of the PACAP/PAC1 pathway. Overall, the results of this study strongly demonstrate the potential of exosome-sheathed ROS-responsive nanogel as a drug carrier to enhance anti-perimenopausal depressive efficacy.

### Supplementary Information

**Additional file 1:** **Fig. S1**. Characterization of purified exosomes. A TEM images of exosomes exocytosed from Raw264.7 cells. Scale bar: 200 nm. B representative number and size of exosomes exocytosed from Raw264.7 cells. C Western blotting analysis of exosome markers (Synt1, CD81, CD63, and ALIX) in cell lysate and exosome. D TEM images of dissociation process of HA NGs@exosomes. **Fig. S2**. The effect of PACAP38 and E2 encapsulation on zeta potential of HA NGs and HA NGs@exosomes. Assays were carried out after overnight encapsulation of 100 µM LLKKK18 in a 0.5 mg/ml HA nanogel solution. Formulations were filtered through a pore size of 0.22 μm before analysis. Both parameters were measured in a Malvern Zetasizer. **Fig. S3**. Colocalization of CD31-Endothelium and CY5.5-HA NGs@exosomes. A images in HA NGs group and HA NGs@exosomes group were detected by confocal microscopy. Scale bar: 50 μm. B the quantification of the fluorescence intensity. Data were presented as mean ± SD for three independent assays. ***P < 0.001. **Fig. S4**. Establishment of OVX + CUMS model. A sucrose preference of mice in control and OVX-CUMS group. B gross anatomy pictures showing the uterus changes in each group. Data were presented as mean ± SD (n = 10 for control, n = 42 for OVX + CUMS group). ##P < 0.01 vs. control. **Fig. S5**. The effect of HA NGs@exosomes on ROS level. A the levels of ROS in each group were tested in mPFC and vHPC. The ROS level in mPFC region (b) and vHPC region (c) were qualified in each group. Data were presented as mean ± SD. ##P < 0.01 vs. control; *P < 0.05, **P < 0.01 vs. OVX-CUMS. **Fig. S6**. The effect of HA NG@exosomes on cytokines and ROS in the LPS-induced neuroinflammatory challenges model. a-c the level of TNF-α, IL-6, IL-1β in each group. Data were presented as mean ± SD (n = 3). ##P < 0.01 vs. control; **P < 0.01 vs. model. **Fig. S7**. The effect of HA NG@exosomes on the expression of key proteins involved in the PACAP/PAC1 receptor pathway in the LPS-induced neuroinflammatory challenges model. The expression of PAC1, PKA, p-CREB, CREB, BDNF were determined by western blot (a) and further quantified (b-e). Data were presented as mean ± SD (n = 3). ##P < 0.01 vs. control; *P < 0.05, **P < 0.01 vs. Model. **Fig. S8.** The effect of HA NG@exosomes on neural synaptic plasticity in the LPS-induced neuroinflammatory challenges model. **a** Immunofluorescence staining of PSD95 and MAP2 was shown. Scale bar: 10 μm. **b** Relative intensity of PSD95 was quantified. Data were presented as mean ± SD (n = 3). ##*P* < 0.01 vs. Control; **P < 0.01 vs. Model.

## Data Availability

The data that support the findings of this study are available from the corresponding author upon reasonable request.

## Abbreviations

ALT: aminotransferase
AT: 11-Amino-1-undecanethiol
BBB: blood-brain barrier
BUN: urea nitrogen
CM: conditioned media
CNS: central nervous system
CUMS: chronic unpredictable mild stress
CRE: serum creatinine
DLS: dynamic light scattering
DMEM: Dulbecco’s Modified Eagle Medium
DMSO: Dimethyl sulfoxide
E2: estradiol
EE: encapsulation efficiency
ELISA: Enzyme-linked immunosorbent assay
FBS: fetal bovine serum
fEPSPs: Field excitatory postsynaptic potentials
FST: Forced swimming test
GSH: Glutathione
HA: hyaluronic acid
LC: loading capacity
LPO: Lipid peroxidation
LTP: long-term potentiation
mPFC: medial prefrontal cortex
NG: nanogel
NHS: N-hydroxysulfosuccinimide
PAC1R: PACAP receptor type 1
PACAP: Pituitary adenylate cyclase-activating polypeptide
PFA: paraformaldehyde
PLT: platelet
PSD95: postsynaptic density protein-95
ROS: reactive oxygen species
SCF: cerebrospinal fluid
SD: standard deviation
SSRIs: selective serotonin reuptake inhibitor
TBIL: total bilirubin
TEM: transmission electron microscopy
TP: total protein
TST: tail suspension test
vHPC: ventral hippocampus
