# Supplementary material for: Exosome-sheathed ROS-responsive nanogel to improve targeted therapy in perimenopausal depression
Source: J Nanobiotechnology. 2023 Aug 8;21:261. doi: 10.1186/s12951-023-02005-y (PMC10408189; doi:10.1186/s12951-023-02005-y)
Supplement: Supplementary file 1 — Additional file 1: Fig. S1. Characterization of purified exosomes. A TEM images of exosomes exocytosed from Raw264.7 cells. Scale bar: 200 nm. B representative number and size of exosomes exocytosed from Raw264.7 cells. C Western blotting analysis of exosome markers (Synt1, CD81, CD63, and ALIX) in cell lysate and exosome. D TEM images of dissociation process of HA NGs@exosomes. Fig. S2. The effect of PACAP38 and E2 encapsulation on zeta potential of HA NGs and HA NGs@exosomes. Assays were carried out after overnight encapsulation of 100 µM LLKKK18 in a 0.5 mg/ml HA nanogel solution. Formulations were filtered through a pore size of 0.22 μm before analysis. Both parameters were measured in a Malvern Zetasizer. Fig. S3. Colocalization of CD31-Endothelium and CY5.5-HA NGs@exosomes. A images in HA NGs group and HA NGs@exosomes group were detected by confocal microscopy. Scale bar: 50 μm. B the quantification of the fluorescence intensity. Data were presented as mean ± SD for three independent assays. ***P < 0.001. Fig. S4. Establishment of OVX + CUMS model. A sucrose preference of mice in control and OVX-CUMS group. B gross anatomy pictures showing the uterus changes in each group. Data were presented as mean ± SD (n = 10 for control, n = 42 for OVX + CUMS group). ##P < 0.01 vs. control. Fig. S5. The effect of HA NGs@exosomes on ROS level. A the levels of ROS in each group were tested in mPFC and vHPC. The ROS level in mPFC region (b) and vHPC region (c) were qualified in each group. Data were presented as mean ± SD. ##P < 0.01 vs. control; *P < 0.05, **P < 0.01 vs. OVX-CUMS. Fig. S6. The effect of HA NG@exosomes on cytokines and ROS in the LPS-induced neuroinflammatory challenges model. a-c the level of TNF-α, IL-6, IL-1β in each group. Data were presented as mean ± SD (n = 3). ##P < 0.01 vs. control; **P < 0.01 vs. model. Fig. S7. The effect of HA NG@exosomes on the expression of key proteins involved in the PACAP/PAC1 receptor pathway in the LPS-induced neuroinflamm [file 12951_2023_2005_MOESM1_ESM.docx]

**Supporting Information 1**

**
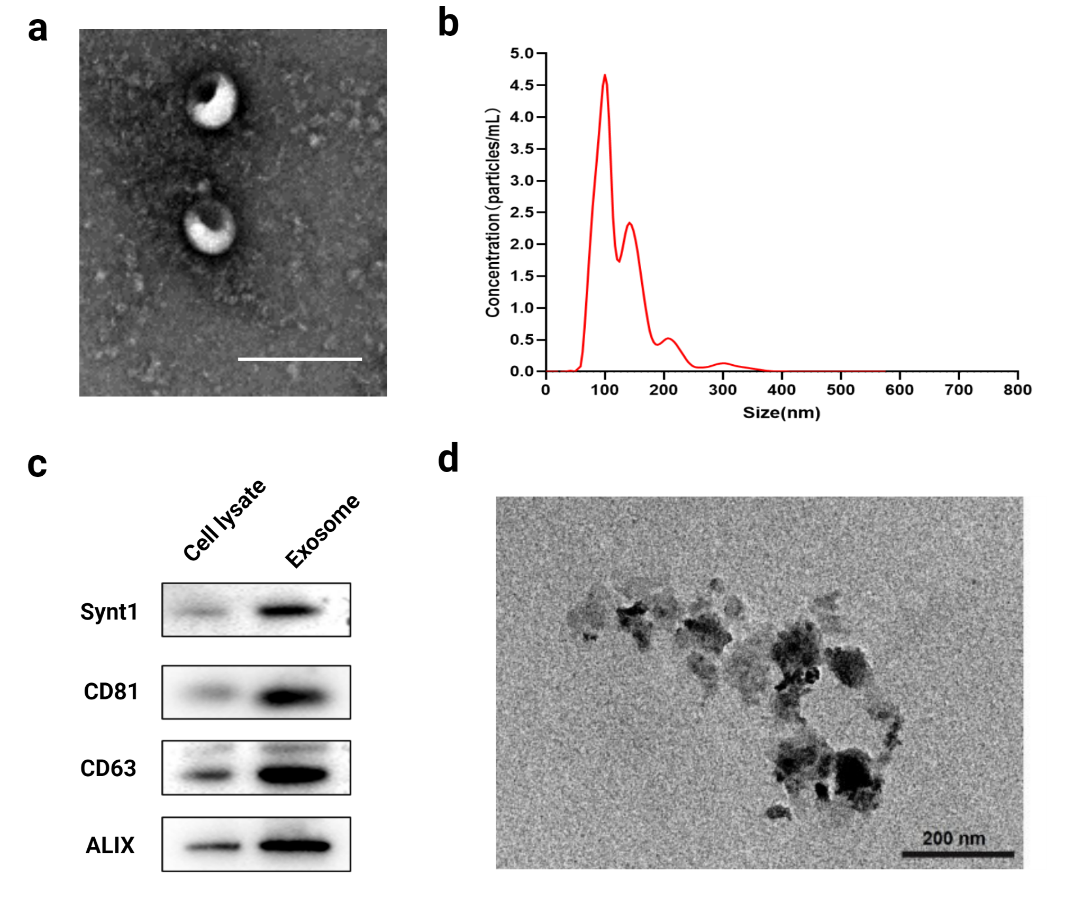
**

**Figure S1** Characterization of purified exosomes. **a** TEM images of exosomes exocytosed from Raw264.7 cells. Scale bar: 200 nm. **b** Representative number and size of exosomes exocytosed from Raw264.7 cells. **c** Western blotting analysis of exosome markers (Synt1, CD81, CD63, and ALIX) in cell lysate and exosome. **d** TEM images of dissociation process of HA NGs@exosomes.

**
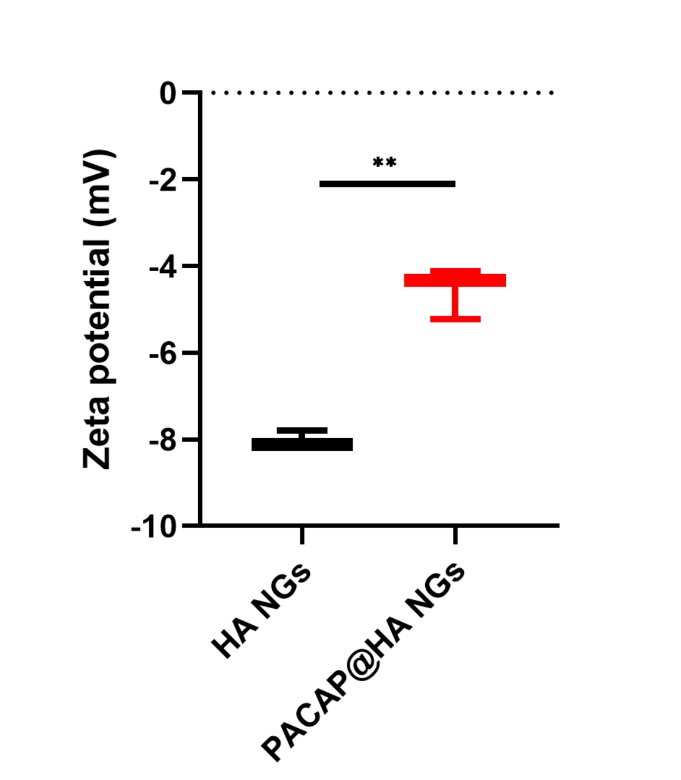
**

**Figure S2** The effect of PACAP38 and E2 encapsulation on zeta potential of HA NGs and HA NGs@exosomes. Assays were carried out after overnight encapsulation of 100 μM LLKKK18 in a 0.5 mg/ml HA nanogel solution. Formulations were filtered through a pore size of 0.22 μm before analysis. Both parameters were measured in a Malvern Zetasizer.

**
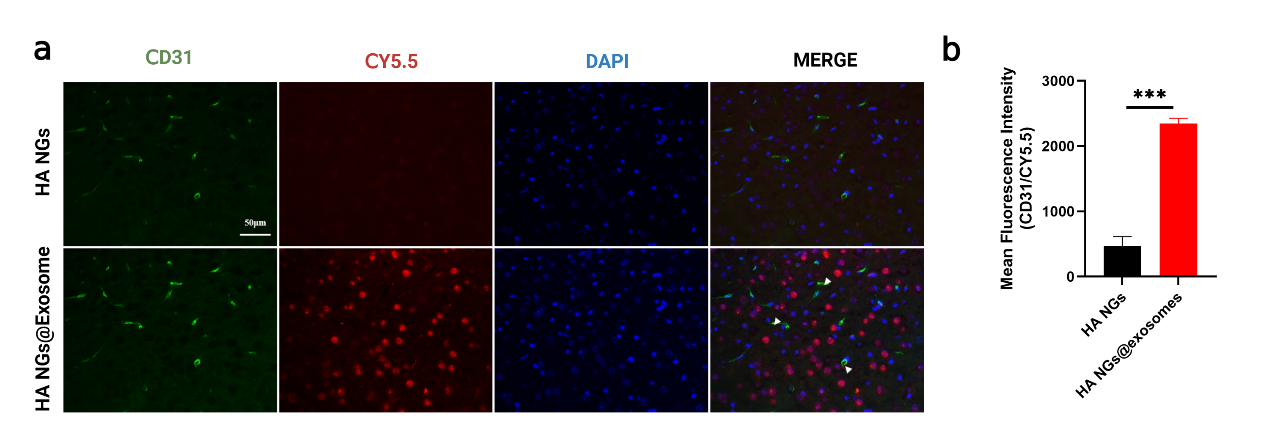
**

**Figure S3** Colocalization of CD31-Endothelium and CY5.5-HA NGs@exosomes. **a** Images in HA NGs group and HA NGs@exosomes group were detected by confocal microscopy. Scale bar: 50μm. **b** The quantification of the fluorescence intensity. Data were presented as mean ± SD for three independent assays. ****P* < 0.001.

**
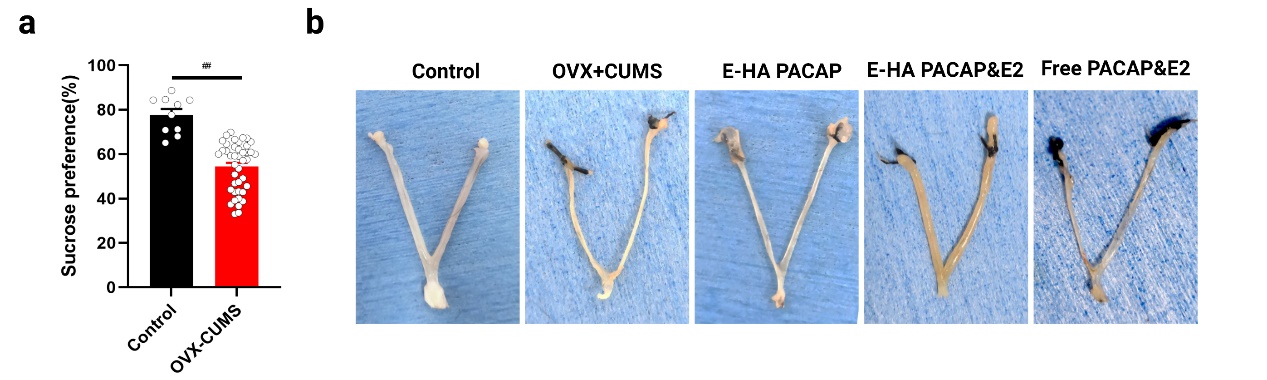
**

**Figure S4** Establishment of OVX+CUMS model. **a** Sucrose preference of mice in control and OVX-CUMS group. **b** Gross anatomy pictures showing the uterus changes in each group. Data were presented as mean ± SD (n=10 for control, n=42 for OVX+CUMS group). ##*P* < 0.01 vs Control.


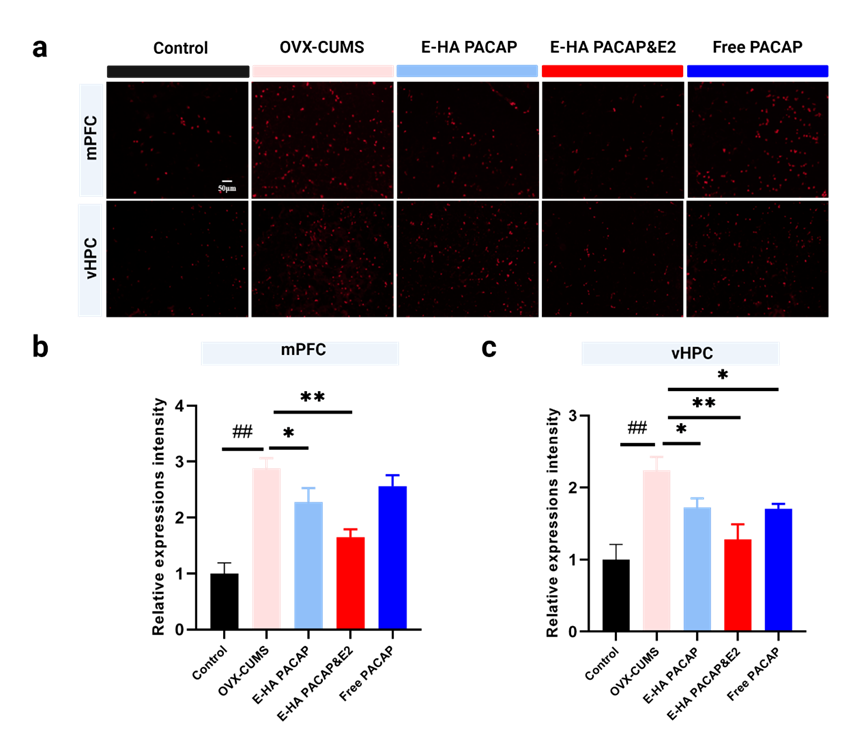


**Figure S5** The effect of HA NGs@exosomes on ROS level. **a** The levels of ROS in each group were tested in mPFC and vHPC. The ROS level in mPFC region (**b**) and vHPC region (**c**) were qualified in each group. Data were presented as mean ± SD. ##*P* < 0.01 vs Control; **P* < 0.05, ***P* < 0.01 vs OVX-CUMS.


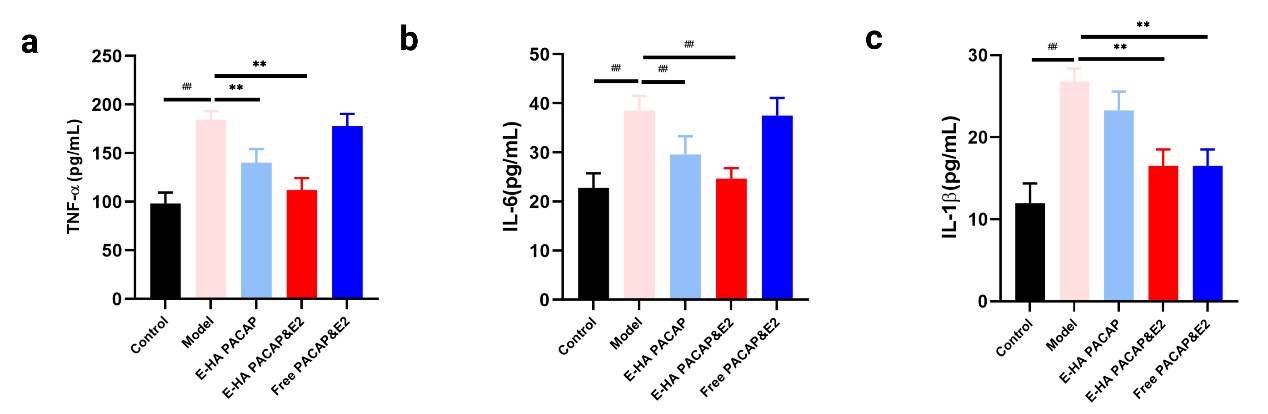


**Figure S6** The effect of HA NG@exosomes on cytokines and ROS in the LPS-induced neuroinflammatory challenges model. **a-c** The level of TNF-α, IL-6, IL-1β in each group. Data were presented as mean ± SD (n=3). ##*P* < 0.01 vs Control; ***P* < 0.01 vs Model.


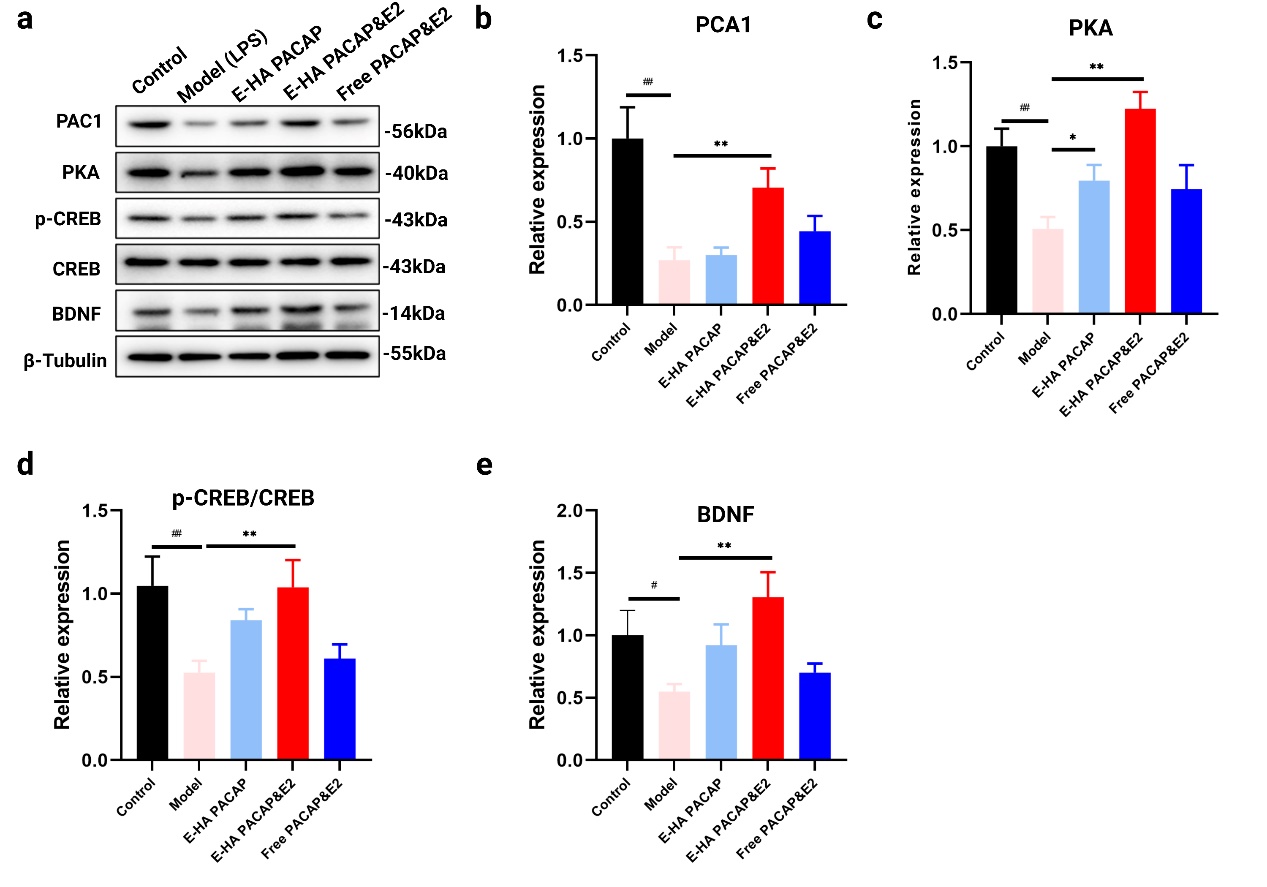


**Figure S7** The effect of HA NG@exosomes on the expression of key proteins involved in the PACAP/PAC1 receptor pathway in the LPS-induced neuroinflammatory challenges model. The expression of PAC1, PKA, p-CREB, CREB, BDNF were determined by western blot (**a**) and further quantified (**b-e**). Data were presented as mean ± SD (n=3). ##*P* < 0.01 vs Control; **P* < 0.05, ***P* < 0.01 vs Model.


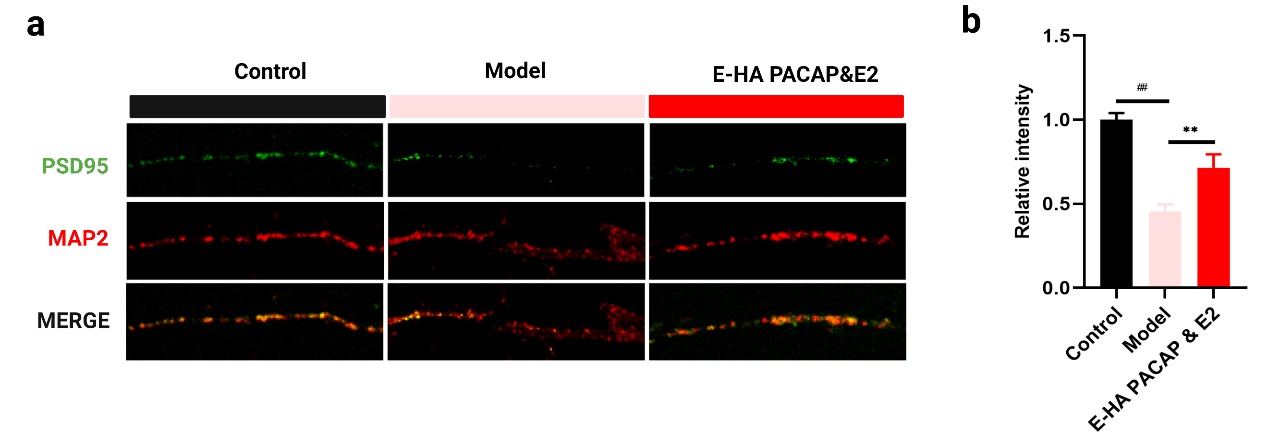


**Figure S8** The effect of HA NG@exosomes on neural synaptic plasticity in the LPS-induced neuroinflammatory challenges model. **a** Immunofluorescence staining of PSD95 and MAP2 was shown. Scale bar: 10 μm. **b** Relative intensity of PSD95 was quantified. Data were presented as mean ± SD (n=3). ##*P* < 0.01 vs Control; ***P* < 0.01 vs Model.
